# Exploratory and Exploitative Innovation Performance in the Artificial Intelligence Industry in China from the Perspective of a Collaboration Network: A Data-Driven Analysis

**DOI:** 10.3390/e27060577

**Published:** 2025-05-29

**Authors:** Liping Zhang, Hailin Li, Wenhao Zhou, Hanhui Qiu, Yenchun Jim Wu

**Affiliations:** 1 College of Business Administration, Huaqiao University, Quanzhou 362021, Fujian, China; zhanglp@hqu.edu.cn (L.Z.); hailin@hqu.edu.cn (H.L.); wenhaoz2021@stu.hqu.edu.cn (W.Z.); 2016411039@stu.hqu.edu.cn (H.Q.); 2 Graduate Institute of Global Business and Strategy, National Taiwan Normal University, Taipei 106, Taiwan; 3 Hospitality Management, Ming Chuan University, Taipei 111, Taiwan

**Keywords:** exploratory innovation performance, exploitative innovation performance, collaboration network, machine learning algorithms, information entropy

## Abstract

Identifying the structural characteristics of a collaboration network that influence firms’ exploratory and exploitative innovation performance can help these firms enhance the output of innovation achievements and their core competitiveness. Based on 14,790 issued patents of 281 firms in the artificial intelligence industry in China, this study explores the complex nonlinear relationship between the structural characteristics of inter-organizational collaboration networks and firms’ exploratory and exploitative innovation performance by using clustering algorithms and classifications based on information entropy or the Gini index. The results indicate the following: (1) The four characteristics of degree centrality, closeness centrality, the local clustering coefficient, and structural holes affect the exploratory and exploitative innovation performance of firms. (2) In different firm clusters, there are different characteristic combinations that provide firms with various development strategies to improve this performance. (3) There are different paths that firms can take to improve this performance, which should be comprehensively considered along with the development goals of firms.

## 1. Introduction

A single firm rarely possesses all the resources required for innovation, and it is challenging to keep up with the pace of technological change by relying solely on its internal capabilities [1]. Therefore, firms need to collaborate with external partners to obtain heterogeneous knowledge resources in order to break through their own technical difficulties, solve core problems in the process of technological innovation, and achieve collaborative innovation [2]. Emerging from Wernerfelt’s seminal work [3], the Resource-Based View postulates that sustainable competitive advantage stems from an organization’s possession of heterogeneous resources characterized by scarcity and non-substitutability [4]. Subsequent scholarly developments have expanded this framework by demonstrating that innovation can be achieved through both internal knowledge refinement and strategic external resource acquisition. Social Network Theory posits that an enterprise’s network centrality, tie strength, and structural configuration collectively determine its capacity for knowledge flow and resource mobilization [5]. As described, the external collaboration network formed by the partnership among different innovation subjects has become important for modern firms in order to carry out innovation activities [6]. The collaboration network has also become one of the important innovation modes widely adopted by global firms [7]. Determining how firms can construct an effective external collaboration network to enhance their technological innovation capacity and performance has become an important problem.

According to the source of knowledge involved in the process of firms’ innovation, it can be divided into two categories. Exploratory innovation is when a firm engages in innovative behavior that deviates from its current knowledge trajectory in order to introduce new products to the market [8]. Exploitative innovation is an innovative behavior that focuses on improving existing knowledge and meeting the current market’s demands [9]. There are significant differences between these two categories in terms of knowledge base and innovation motivation, and they also have different impacts on firms. In general, exploratory innovation broadens a firm’s existing knowledge base, while exploitative innovation deepens the understanding and utilization of the firm’s knowledge base [10].

From a Resource-Based View perspective, exploratory innovation necessitates the organizational transcendence of existing resource boundaries, whereas exploitative innovation requires intensive refinement of extant knowledge repositories [11]. Social Network Theory elucidates distinct governance mechanisms for these innovation paradigms: exploratory initiatives demand open-network configurations to facilitate novel knowledge recombination, while exploitative processes thrive in closed-network architectures that enable knowledge exploitation efficiency [12]. Clearly, the differences between the two innovation models in terms of knowledge base, collaboration network structure, and innovation motivation essentially reflect the differentiated allocation strategies of firms for network resources and internal resources: exploratory innovation builds a first-mover advantage by broadening the knowledge base to explore new markets, while exploitable innovation consolidates the existing market share by deepening the application of knowledge. Exploratory and exploitative innovation are significantly important to the long-term survival and development of firms. Improving their performance has become an important development direction in the process of firms’ technological innovation.

The structure of a collaboration network affects the efficiency of knowledge and resources among its members and has a significant impact on a firm’s innovation performance [13]. Based on the Resource-Based View and Social Network Theory, in order to reveal the impact of the structural characteristics of the external collaboration network on firms’ exploratory and exploitative innovation performance, this study takes 281 firms in China’s artificial intelligence industry as the research sample, and systematically discusses the combination of key influencing factors affecting the exploratory and exploitative innovation performance of firms, providing a theoretical basis for firms to construct a collaborative network mechanism adapted to different innovation goals. According to relevant research on innovation management problems [14], the social network analysis (SNA) method [15] and machine learning algorithms, such as hierarchical clustering [16] and classification and regression tree (CART) [17], are used to answer the following questions: (1) What are the similarities and differences in the structural characteristics of the collaboration network of firms in different clusters? (2) Which structural characteristics of this network will affect the exploratory and exploitative innovation performance of firms in different clusters? (3) How do we improve the exploratory and exploitative innovation performance of different types of firms by adjusting the structural characteristics of the collaboration network?

This study uses machine learning algorithms to analyze and discuss factors that affect this performance. It can not only deeply mine valuable knowledge and management rules, open the “black box” of the complex relationship structural among variables, and enrich the theoretical research in related fields, but also provide a theoretical reference for the practice of firms’ innovation development and the implementation of innovation-driven policies in industries.

The subsequent sections of this paper are organized as follows. Section 2 provides a comprehensive review of the existing research that synthesizes key findings and theoretical perspectives from the relevant research and presents the theoretical model. Section 3 presents the research framework and methodologies including the hierarchical clustering algorithm and decision tree classification. Section 4 introduces the selection and measurement of variables. Section 5 introduces data acquisition and processing and further divides firms into three clusters. Section 6 analyzes the decision rules of three firm clusters for Exploratory and Exploitative Innovation Performance. Section 7 summarizes the conclusions and discusses the theoretical contributions, managerial implications, limitations, and future research.

## 2. Literature Review

To conduct an in-depth investigation into the mechanisms by which collaboration networks influence firms’ exploratory and exploitative innovation performance, this study systematically reviewed and synthesized the existing relevant research findings.

### 2.1. Exploratory and Exploitative Innovation Performance

According to March [18], innovation can be categorized into exploratory and exploitative, exhibiting significant differences in the required resources, risk profiles, and potential returns. Exploratory innovation emphasizes the acquisition and creation of novel knowledge and technologies, representing an outward-looking innovation mode where firms seek external knowledge and technological breakthroughs [19]. Exploitative innovation, however, prioritizes the refinement, integration, and improvement of existing knowledge and technologies, constituting a reformative innovation behavior [20].

Numerous studies have examined the heterogeneous effects of various influencing factors on exploratory and exploitative innovation performance from both internal and external perspectives. From an internal firm perspective, Xu et al. [21] investigated the differential impact of executives on innovation, finding that environmental policy uncertainty within firms enhances exploitative green innovation performance but has no significant effect on exploratory innovation. Ouyang et al. [22] explored how reverse knowledge transfer from subsidiaries to parent companies influences innovation, revealing that manufacturing-related internal knowledge primarily boosts exploitative innovation, while externally sourced knowledge related to product development and sales is more conducive to exploratory innovation. Enkel et al. [23] demonstrated that employees’ knowledge absorption capacity positively affects exploratory innovation performance but shows no significant impact on exploitative innovation. Meanwhile, the external environment has also been a focal point in studying the differential effects on these two innovation types. For instance, Liu et al. [24] analyzed how policy environments distinctly influence exploratory and exploitative innovation performance, showing that government subsidies promote exploratory green innovation, whereas pollution fees incentivize firms to pursue exploitative green innovation. Bachmann et al. [25] observed that firms facing intense market competition tend to prioritize exploratory innovation, while entering emerging markets more strongly motivates small-scale firms to engage in exploitative innovation. Shukla et al. [26] emphasized that broader partner alliances enhance exploratory innovation performance but hinder exploitative innovation, whereas deeper alliances yield the opposite effect—greater depth is more likely to lead to an improvement in exploitative innovation performance and reduce the outcomes of exploratory innovation.

### 2.2. Collaboration Network and Exploratory and Exploitative Innovation Performance

The collaboration network is an important factor influencing the innovation performance of firms. Social network analysis is a methodology used to study the relationships between individuals or organizations in a network and their spatial structure based on relational data [27]. Many studies have demonstrated that the location and structure of innovation members in the collaboration network can significantly impact a firm’s innovation performance by influencing their ability to acquire resources and information, as well as their ability to cope with risks [28]. Numerous studies have investigated the impact of network structure heterogeneity on exploratory and exploitative innovation performance.

The current analysis of network characteristics is typically divided into global and individual characteristics. Global network characteristics include network size and network density, among others [29]. Many studies have generated valuable insights. However, there are still significant controversies. For instance, Ponta et al. [30] discovered that having a vast network of patent collaborations can enhance the efficiency of continuous innovation. However, Min et al. [31] identified an interactive effect between R&D investment and network scale, demonstrating that elevated R&D investment levels can compensate for a constrained network scale to achieve enhanced innovation efficiency. Sime et al. [32] suggested that a higher network density indicates a closer relationship between network members, making it easier for members to access resources, which can lead to improved innovation performance. However, Zhao and Li [33] found an inverted U-shaped relationship between network density and innovation performance. They believe that excessive network density will result in regional information homogenization, thus affecting the research and development intentions of network members. The individual characteristics that signify the location of members within a network can significantly impact their innovation performance [34]. One of the key individual characteristics within a network is centrality [35]. Researchers typically concentrate on three types of centrality: degree centrality, closeness centrality, and betweenness centrality [28]. Di et al. [36] revealed that degree centrality and structural holes are crucial in determining a firm’s position within the network, and firms situated in the core position of the network tend to have better production efficiency, which translates into higher levels of innovation performance. Kang et al. [37], in their research on the hydropower industry, found that firms with high degree centrality and more structural holes are more likely to overcome regional limitations and achieve higher innovation performance. Chen et al. [38] showed that high closeness centrality promotes exploratory innovation but does not significantly influence exploitative innovation. Ju et al. [11] emphasized that strong ties in collaboration networks facilitated the generation of exploratory innovation; however, they exhibited no significant impact on exploitative innovation. Guan et al. [39] postulated an inverted U-shaped relationship between direct ties and both innovation types, while revealing that indirect ties positively influenced exploratory innovation performance but demonstrated negligible effects on exploitative innovation performance.

Concurrently, existing research has bifurcated into intra-organizational versus inter-organizational dimensions to dissect the impacts of the heterogeneous innovation of collaboration networks: Reagans and Zuckerman [40] posited that dense internal networks accelerated organizational knowledge sharing, thereby catalyzing innovation performance enhancement. Xie et al. [41] demonstrated that open intra-organizational networks with superior knowledge absorptive capacity disproportionately benefited radical innovation outcomes. Van der Wal [42] established that tightly knit networks fostered trust-mediated information exchange mechanisms essential for innovation genesis. Xiao et al. [43] revealed the paradox of structural holes: greater network brokerage opportunities facilitated heterogeneous knowledge acquisition and recombinant innovation potential. In addition, the impact of the organization’s external collaboration network on innovation performance was also a focus of scholar attention: Pan et al. [44] identified a positional advantage: centrally positioned firms leveraged network resources to exploit unique market opportunities, significantly boosting exploratory innovation. Li et al. [5] uncovered closure benefits: cohesive external networks enhanced innovation through redundant yet reliable knowledge channels. Wen et al. [45] emphasized that both exploratory and exploitative innovation could help firms obtain sustainable competitive advantages and, in the inter-firm R&D collaboration network, structural holes moderated the effect of network knowledge diversity on firms’ exploitative and exploratory innovation outcomes.

Based on the above analysis, many scholars have explored the impact of the collaboration network on the innovation performance of firms. However, current research primarily employs empirical analytical methods, such as regression analysis, focusing mainly on the linear or simple nonlinear effects of collaboration network characteristics on firms’ innovation performance. Few studies have investigated the complex nonlinear effects of multiple collaboration network characteristics and their combinations on exploratory and exploitative innovation performance. Furthermore, most existing studies overlook the heterogeneous characteristics of firms, adopting a generic perspective to analyze the influencing factors of exploratory and exploitative innovation performance. This approach makes it difficult to derive targeted research conclusions and managerial implications. Therefore, there is an urgent need to adopt data-driven analytical methods to reveal, based on objective data, the complex mechanisms through which the structural characteristics of collaboration networks and their combinations affect exploratory and exploitative innovation performance across different types of firms. This will provide differentiated managerial insights for firms to enhance both forms of innovation performance, while offering new research perspectives and theoretical references for enriching corporate innovation management theory.

### 2.3. Theoretical Model

Indeed, the relationship between collaboration network structural characteristics and firms’ exploratory and exploitative innovation performance is unlikely to be a simple linear correlation, but rather to exhibit complex interdependencies. For instance, the impact of structural holes on exploratory innovation performance may depend on a firm’s degree centrality; high degree centrality may amplify the benefits of structural holes by enabling efficient access to diverse knowledge, while low degree centrality may limit effectiveness. Furthermore, the combined effects of multiple structural characteristics of collaboration networks, such as the local clustering coefficient interacting with structural holes, may have heterogeneous effects. When the local clustering coefficient is high, firms are more likely to be exposed to homogeneous knowledge resources, which may hinder the improvement in exploratory innovation performance. Conversely, when the local clustering coefficient is low, firms with greater structural hole advantages can acquire more heterogeneous knowledge, leading to higher exploitative innovation performance. Consequently, understanding how the structural characteristics of collaboration networks influence exploratory and exploitative innovation performance requires analyzing these multifaceted interactions and complex mechanisms.

**Figure 1:**
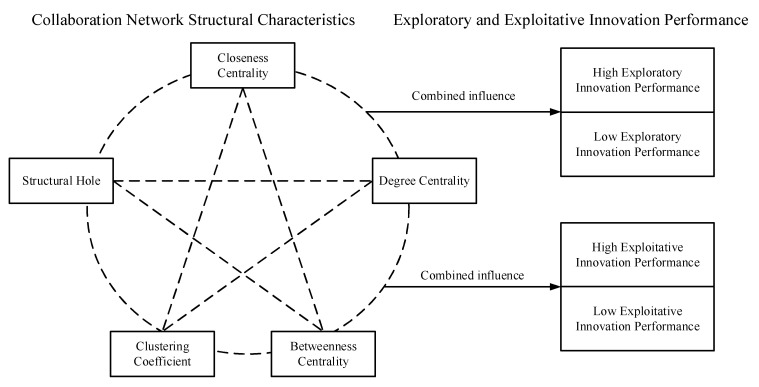
Theoretical model.

This study examines the complex nonlinear influence of five structural characteristics of collaboration networks on both firms’ exploratory and exploitative innovation performance, aiming to reveal their complex mechanisms. The findings will provide practical guidance for firms to stimulate and enhance their exploratory and exploitative innovation performance, by strategically optimizing their network positions. The theoretical framework is presented in Figure 1.

## 3. Research Design

In this section, both the hierarchical clustering algorithm and CART algorithm are introduced. Meanwhile, the research framework is also proposed.

### 3.1. Research Framework

**Figure 2:**
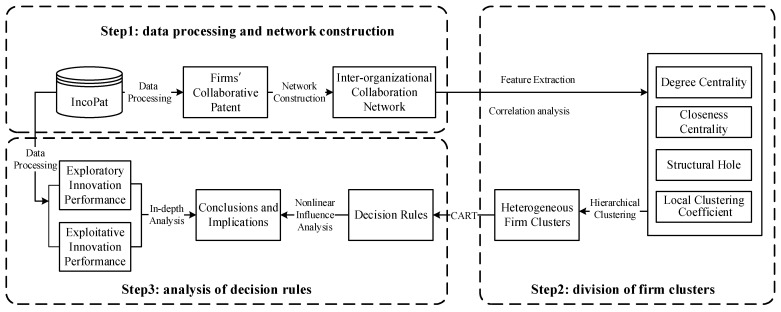
Research framework.

In this study, a research framework with machine learning algorithms was constructed to explore the complex relationship between the structural characteristics of the collaboration network and firms’ exploratory and exploitative innovation performance. As shown in Figure 2, the original patent data from the incoPat global patent database were selected as the data source, and the related firms and their corresponding issued patents were obtained through data processing. Based on the collaborative relationship between firms and other firms, universities, research institutions, and other external organizations, an inter-organizational collaboration network was constructed, and the key structural characteristics of the collaboration network were further selected by combining the feature extraction and correlation analysis. According to the difference in variable characteristics, 281 firms were accurately divided by the hierarchical clustering algorithm, and each firm cluster was analyzed. Regarding the structural characteristics of the collaboration network as conditional attributes and the level of exploratory and exploitative innovation performance of firms as the decision attributes, the CART algorithm was employed to conduct an in-depth mining and analysis of decision rules that affect the exploratory and exploitative innovation performance of firms in different clusters. Finally, the complex nonlinear relationship between the collaboration network structural characteristics and firms’ exploratory and exploitative innovation performance is further discussed.

### 3.2. Research Methodology

*Hierarchical clustering algorithm.* This [16] is a clustering algorithm of unsupervised learning. By clustering the given unlabeled training samples with this algorithm, the sample in the dataset can be divided into several disjoint subsets [17]. Each subset is called a cluster. The members of the same cluster are the most similar, while members of different clusters are the least similar. The principle of the agglomerative hierarchical clustering algorithm is simple and easy to implement [46]. First, each sample forms a cluster on its own. Then, distances between all clusters are calculated. Clusters with the smallest distances are iteratively merged together until all samples are in the same cluster. Alternatively, the final clustering result does not change, due to the preset number of clusters, so objectivity is an advantage of this algorithm. In view of the simple principle and easy implementation of the hierarchical clustering algorithm, this study used this algorithm to divide firms with similar structural characteristics of the collaboration network into the same cluster.

*Decision Tree Classification.* Decision trees represent a significant classification and prediction methodology, serving as predictive models that effectively capture the mapping relationships between object attributes and their corresponding values [47]. Valued for their interpretability [48], decision trees employ a tree-like graphical structure to visually represent the sequential decision-making process. This structure can be readily transformed into classification rules, which essentially represent association rules among features or variables. Such rules are particularly effective in uncovering relationships between variables and identifying potential decision pathways leading to specific outcomes. Consequently, decision tree algorithms prove highly suitable for analyzing interactive effects among influencing factors. Within the current domain of management research, an increasing number of scholars have adopted data-driven approaches utilizing decision tree algorithms to investigate nonlinear relationships and complex influence mechanisms among variables. For instance, Grant and Yeo [49] employed the CHAID decision tree algorithm to analyze factors and pathways influencing firm performance. Zhou and Li [50] applied the CART algorithm to examine nonlinear relationships and complex mechanisms between multiple influencing factors and breakthrough innovation. Similarly, Li and Tian [51] utilized the CART algorithm from a TOE perspective to analyze the complex interactive effects of multiple factors on corporate digital technology innovation.

Among the prominent algorithms, Iterative Dichotomiser 3 (ID3) and Classification and Regression Trees (CARTs) adopt distinct approaches to feature selection and tree construction [52]. The ID3 algorithm, for instance, uses information entropy to measure data purity and select splitting attributes. For a sample set D, the entropy Ent(D) is defined as follows:

(1) $$Ent(D)=-\sum_{i=1}^{m}p_{i}\log_{2}p_{i},$$

where $p_{i}$ is the proportion of class i in D, and m is the number of distinct classes in D. Higher entropy indicates greater impurity, meaning that the dataset contains a more uniform distribution of classes. Conversely, lower entropy signifies higher purity, where one class dominates. In the ID3 algorithm, information gain is used to evaluate the effectiveness of a splitting attribute. It measures the reduction in entropy after partitioning the dataset by a specific attribute. The information gain of attribute A is calculated as

(2) $$Gain(A)=Ent(D)-Ent_{A}(D),$$

where $Ent_{A}(D)$ is the weighted entropy after partitioning D by attribute A:

(3) $$Ent_{A}(D)=\sum_{j=1}^{v}\frac{\left|D_{j}\right|}{\left|D\right|}Ent(D_{j}),$$

where $D_{j}$ represents the subset of D where attribute A takes the j−th value, and v is the number of possible values for A. While effective for categorical data, ID3 tends to overfit and cannot handle continuous features directly.

CART employs the Gini index to evaluate impurity:

(4) $$Gini(D)=1-\sum_{i=1}^{m}p_{i}^{2}.$$

A lower Gini index reflects greater purity within the data. The CART algorithm recursively partitions the data to minimize Gini impurity, producing a binary tree with clear decision rules. Considering the CART algorithm’s distinctive advantages in effectively handling continuous variables and demonstrating robustness to outliers, along with its binary tree structure that generates relatively few yet highly interpretable branches, this study employed this method to construct decision trees for each firm cluster based on previous research. Through this approach, we extracted decision rules to explore potential decision pathways for firms to achieve both exploratory and exploitative innovation performance. Furthermore, we conducted an in-depth analysis of the interaction effects among collaborative network structural characteristics influencing exploratory and exploitative innovation performance, as well as the complex influence mechanisms between variables.

## 4. Selection and Measurement of Variables

In order to analyze the influence of a collaboration network on firms’ exploratory and exploitative performance, it is necessary to select and measure suitable collaboration network structural characteristics and firms’ exploratory and exploitative innovation performance. Based on the literature review, degree centrality, betweenness centrality, closeness centrality, the local clustering coefficient, and structural holes from the structural characteristics of the collaboration network were selected as independent variables to investigate their impacts on both the exploratory and exploitative innovation performance of firms. This section introduces the detailed measurement of the variables based on the existing studies.

*Degree Centrality.* In general, the degree centrality (*DC*) of a network is often represented by the node degree. We define the degree of network node *i* as

(5) $$DC_{i}=\sum_{j=1}^{n}X_{ji},$$

where *n* denotes the number of network nodes. When node *j* is connected to node *i*, $X_{ji}=1$. Otherwise, $X_{ji}=0$.

*Betweenness Centrality.* The betweenness centrality (*BC*) of a network refers to the degree to which one node in the network is located at the center of other nodes. A firm’s central position in the collaboration network can promote its innovation output. The specific calculation formula of *BC* is

(6) $$BC_{i}=\sum_{j\neq k}g_{jk}\left(n_{i}\right)/g_{jk},$$

where $g_{jk}\left(n_{i}\right)$ refers to the number of shortest paths between two nodes *j* and *k* through node *i*. $g_{jk}$ represents the number of shortest paths between two nodes *j* and *k*.

*Closeness Centrality.* The closeness centrality (*CC*) of the network requires measuring the average length of the shortest path from each node to the other nodes. That is, the closer one node is to other nodes, the higher its centrality. In general, for facilities that need to be used by as many people as possible, their *CC* is commonly high. We define the average path length of node *i* in the network as

(7) $$d_{i}=\frac{1}{n-1}\sum_{j\neq i}d_{ij},$$

where $d_{ij}$ refers to the number of shortest paths between two nodes *i* and *j*. *n* denotes the number of nodes in the network. Therefore, the *CC* of node *i* in the network can be defined as

(8) $$CC_{i}=\frac{1}{d_{i}}=\frac{n-1}{\sum_{j\neq i}d_{ij}}.$$

*Clustering Coefficient.* The aggregation coefficient, also known as the clustering coefficient, can be divided into the global clustering coefficient (*GLC*) and the local clustering coefficient (*LCC*). The former can evaluate the aggregation degree of an overall network, while the latter can measure the aggregation degree near one node in the network. Since this study mainly focuses on the influencing factors and mechanisms of the inter-organizational collaboration network on firms’ exploratory and exploitative innovation performance, this study selected the *LCC* as the final aggregation coefficient. Referring to the practice of Fleming et al. [53], the *LCC* of node *i* is defined as

(9) $$LCC_{i}=\frac{2a_{i}}{k_{i}\left(k_{i}-1\right)},$$

where $a_{i}$ denotes the actual number of edges between node *i* and its adjacent points. $k_{i}$ is the degree of node *i*.

*Structural Hole.* The structural hole (*SH*) reflects the degree of redundancy between firm nodes in the network, which pays more attention to the relationship mode between firms that are self-connected. At present, the *LD* is usually applied to measure the *SH*. Since *LD* is a negative indicator, the lower the *LD* of one node, the more it occupies the intermediary position of other nodes, and the stronger its ability to transfer knowledge and obtain information with other nodes [54]. To this end, many researchers have performed various treatments when building the index of *SH*. For example, Guan and Zhang [55] used “2-*LD*” to measure the *SH*. In this study, we chose the *LD* as the measurement of the richness of the *SH* of nodes, and we used “2-*LD*” to describe the density of *SH* in the network. The detailed formula for *SH* is

(10) $$\begin{array}{l} C_{iu}={\left(p_{iu}+\sum_{v\neq i,v\neq u}p_{iv}p_{vu}\right)}^{2}\\ C_{i}=\sum_{u}C_{iu}\\ SH_{i}=2-C_{i} \end{array},$$

where $p_{iu}$ is the direct relationship input of node *i* to node *u*. $p_{iv}$ refers to the intensity ratio of the relationship between node *i* and node *v*. $p_{vu}$ represents the intensity ratio of the relationship between node *v* and node *u*.

*Exploratory Innovation Performance.* This study draws on the practice of Zaefarian et al. [34] and distinguished and measured firms’ exploratory innovation performance (*ERIP*) based on the technology categories represented by the first four digits of the International Patent Classification (*IPC*) number (namely, *IPC* subclass), i.e., taking the technology categories included in all patents of firms issued from 2017 to 2019 as the judgment basis, counting the number of all patents in the new patent technology category that appeared in 2020–2022 but did not exist in 2017–2019, and regarding this number as a firm’s *ERIP*.

*Exploitative Innovation Performance.* Similarly, via the practice of Gilsing et al. [56], this study distinguishes and measures firms’ exploitative innovation performance (*EIIP*) according to the technology category represented by the first four digits of the *IPC* number. That is, this study measures firms’ *EIIP* by subtracting their *ERIP* from the total number of patents issued from 2020 to 2022.

## 5. Data and Division of Firms

In this section, we describe how the original patent data were processed and how the structural characteristics of the inter-organizational collaboration network were selected. Meanwhile, firms with similar structural characteristics were clustered using the hierarchical clustering algorithm.

### 5.1. Data Acquisition and Processing

Due to the highly digital nature of the artificial intelligence industry, many innovation processes can be digitally recorded and tracked, and the data of this emerging industry can reflect current market dynamics and technological advancements [57]. Therefore, in this study, the issued patents in China’s artificial intelligence industry from the incoPat global patent database were selected as the research samples. In view of the lag in the influence of the collaboration network on firms’ exploratory and exploitative innovation performance, referring to the experience and practice of existing research [18], we constructed an inter-organizational collaboration network by using the issued patents from 2017 to 2019, and we calculated firms’ exploratory and exploitative innovation performance by applying the issued patents from 2017 to 2022. After searching for a series of topics related to artificial intelligence, 235,905 patents were found from the incoPat global patent database, containing 52,042 patents from 2017 to 2019 and 183,863 patents from 2020 to 2022. There were 145,184 patents that firms participated, among which there were 13,311 collaborative patents and 131,873 independent patents. To avoid the sparseness of the inter-organizational collaboration network, we selected firms with three or more collaborative patents as the basis in the first three years (2017–2019), and we obtained 281 major firms through name matching with firms in the last three years (2020–2022). In addition, 14,790 patents from the 281 firms were found from 2017 to 2022, including 7897 independent patents and 6893 collaborative patents.

### 5.2. Selection of the Collaboration Network Structural Characteristics

**Figure 3:**
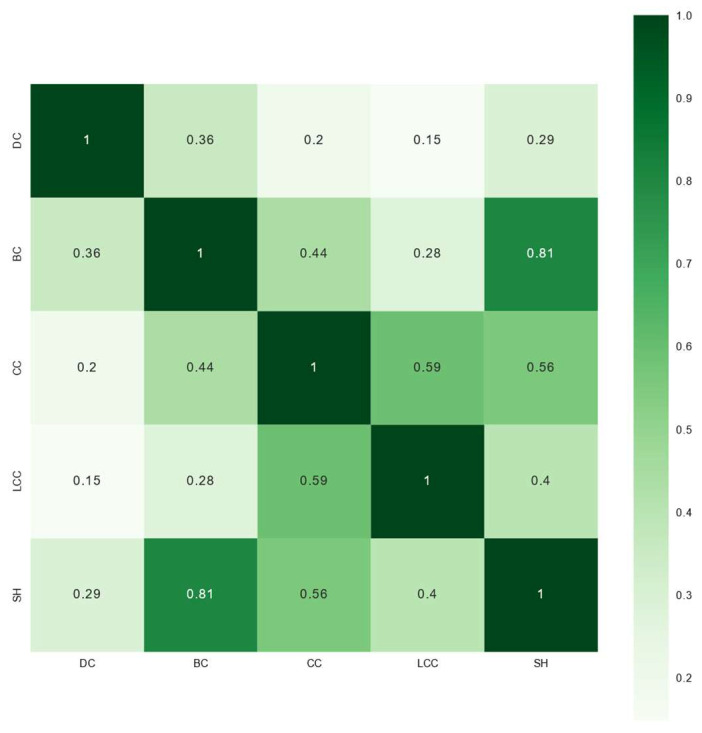
Pearson correlation coefficients between the structural characteristics of the collaboration network.

The collinearity among characteristic variables may affect the acquisition of multiple influencing factors of firms’ exploratory and exploitative innovation performance. The Pearson correlation coefficients (*PCCs*) between the structural characteristics of the collaboration network were obtained as follows. As shown in Figure 3, there are some structural characteristics of the collaboration network that are positively correlated, such as the *SH* and *BC* (*PCC* = 0.81). To eliminate the strong correlation between *SH* and *BC*, it was necessary to delete one of them. Considering that the *SH* focuses on ego networks by emphasizing local topology, which fundamentally differs from *BC*, relying on the global network topology, *SH* is more conducive to identifying critical bridging nodes in the collaboration network [58]. Based on the above calculation formulas, the characteristic variables, such as *DC*, *CC*, *LCC*, and *SH* and firms’ exploratory and exploitative innovation performance (namely, *ERIP* and *EIIP*), were calculated. The maximum Variance Inflation Factor (VIF) among these characteristic variables is 1.894; therefore, it can be considered that there was no significant multicollinearity among the variables.

Hence, four collaboration network structural characteristics, *DC*, *CC*, *LCC*, and *SH,* were selected. Furthermore, to identify firms with similar structural characteristics to the collaboration network and obtain the decision rules for the exploratory and exploitative innovation performance in fine granularity, this study divided the 281 firms into different clusters based on their *DC*, *CC*, *LCC*, and *SH*. The variable data of focal firms can be found in the Supplementary Materials.

### 5.3. Clustering Analysis of Firms

**Figure 4:**
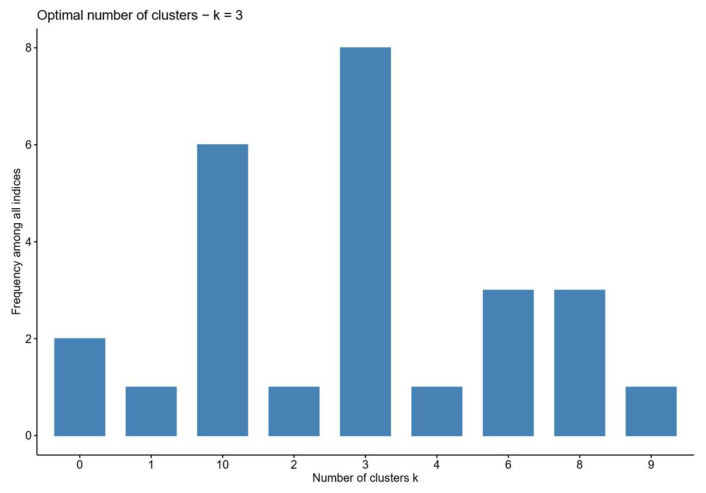
The optimal cluster number.

Before clustering, the Cloud Model was used to transform previously unevenly distributed data, which can consistently translate qualitative concepts into quantitative data [59]. Based on the convenience and interpretation of the hierarchical clustering algorithm, this study used this algorithm with the Euclidean distance to measure the dissimilarity between firms based on their collaboration network structural characteristics and the Ward.D2 linkage method to minimize the total within-cluster variance by merging clusters at each step of the hierarchical process, thereby effectively grouping firms with homogeneous network patterns into distinct clusters. The optimal number of clusters was determined as k = 3 through the NbClust package, which evaluates multiple clustering validity indices, with three clusters receiving the highest number of votes among all candidate solutions, as shown in Figure 4.

**Figure 5:**
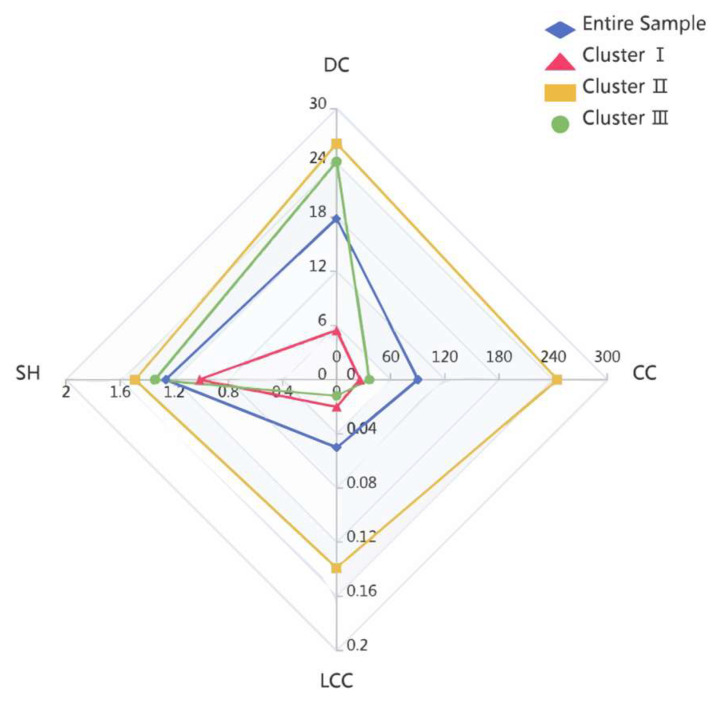
Radar chart of firm clusters based on collaboration network structural characteristics.

Based on the average value of the *DC*, *CC*, *LCC*, and *SH*, three different firm clusters were obtained, as shown in Table 1. Among them, the values of the *DC*, *CC*, *LCC*, and *SH* are the average value of the corresponding collaboration network structural characteristics of each cluster. The letter “H” or “L” in the “Proportion of *ERIP*” and “Proportion of *EIIP*”, respectively, indicate that the level of innovation performance is high or low. To effectively reduce the potential distortion of extreme values on the research results and avoid partition bias inherent in mean-based classification, while ensuring balanced sample sizes for both high- and low-innovation-performance groups, this study drew on the practice of Zhang et al. [60] by employing the median values of the exploratory and exploitative innovation performance of 281 firms to divide the high and low innovation performance. The innovation performance exceeding the median was set to high; otherwise, it was set to low. The detailed radar chart is shown in Figure 5.

The characteristics of three firm clusters presented in Table 1 and Figure 5 indicate the following: (1) There are 103 firms in Cluster I, accounting for 36.7% of all firms. The number of external collaboration partners is small, and firms in this cluster have the least non-redundant connection with other organizations. Compared with the other two firm clusters, the *CC* is at a minimum, which indicates that the average path distance between firms is farthest. Meanwhile, the proportions of firms obtaining high *ERIP* and high *EIIP* in this cluster are both 38.8%, indicating that the output of the exploitative innovation achievements of firms in this cluster are similar to its exploratory innovation achievements. That is, it is equally difficult for firms in this cluster to obtain a high *ERIP* and high *EIIP*. (2) Cluster II contains 78 firms, which account for 27.8% of all firms. Compared with the other two firm clusters, the four characteristics of *DC*, *SH*, *CC*, and *LCC* are all at the maximum. Firms in this cluster have a large number of partners, and they occupy the most structural hole positions and have the most non-redundant connections with external organizations. Furthermore, the relationship between the collaborative partners in this cluster may be closer, and the knowledge exchange may be more frequent. In addition, the proportions of high *ERIP* and high *EIIP* are 62.8 and 43.6%, respectively, indicating that it is easy for firms in this cluster to obtain a high *ERIP*. (3) There are 100 firms in Cluster III, which only account for 35.6% of all firms. Furthermore, the *LCC* of this cluster is at the minimum, indicating that the group formed by neighbor nodes is sparse. Meanwhile, the average distance between the firms in this cluster and their external collaboration partners is far. However, the firms in this cluster have more external collaboration partners as well as more non-redundant connections with external organizations, and the proportions of a high *ERIP* and high *EIIP* are 54.0% and 71.0%, respectively, which indicates that it is more likely for firms in this cluster to obtain high exploitative innovation performance.

## 6. Analysis of Decision Rules

Based on the correlation analysis, the *DC*, *CC*, *LCC*, and *SH* did not have a clear linear or simple nonlinear relationship with firms’ exploratory and exploitative innovation performance, indicating that firms’ exploratory and exploitative innovation performance cannot be determined by a single collaboration network structural characteristic but may be affected by several structural characteristics at the same time. That is, there may be a complex nonlinear relationship between the collaboration network structural characteristics and firms’ exploratory and exploitative innovation performance. The CART algorithm was implemented in Python version 3.9.7 using PyCharm 2020.3.3, with *DC*, *CC*, *LCC*, and *SH* as the conditional attributes and the *ERIP* and *EIIP* of firms as the decision attributes. A comprehensive pruning strategy was adopted, including pre-pruning with max_depth and min_samples_leaf parameters to constrain tree growth and prevent overfitting, followed by post-pruning to remove redundant branches while maintaining model generalizability.

Applying the CART algorithm yielded decision trees and corresponding decision rules for *ERIP* and *EIIP* across the three firm clusters. In the decision rule tables, the support degree refers to the proportion of the sample size supporting the current decision rule in the sample size of the firm cluster. The larger the proportion, the more samples support the current decision rule. The confidence degree represents the proportion of the sample size supporting the current decision rule of the leaf node. The larger the proportion, the more credible the current decision rule. The “high” or “low” for decision results reflects the level of firms’ *ERIP* and *EIIP*, and the level is determined by the median of the *ERIP* and *EIIP* of 281 firms.

From the decision rules in different firm clusters, when the sample data do not obey any distribution, the CART algorithm can be used to explore the fact that multiple structural characteristics of the collaboration network jointly affect firms’ *ERIP* and *EIIP*. This not only avoids the limitations of traditional regression methods on the distribution of the sample data but also reveals the combination effect of multiple characteristic factors affecting firms’ *ERIP* and *EIIP*. Next, we analyzed the detailed decision rules for *ERIP* and *EIIP* in three different firm clusters.

### 6.1. Decision Rules for ERIP

The decision rules for *ERIP* in different firm clusters were analyzed separately.

(1) Decision rules for *ERIP* in Cluster I.

**Figure 6:**
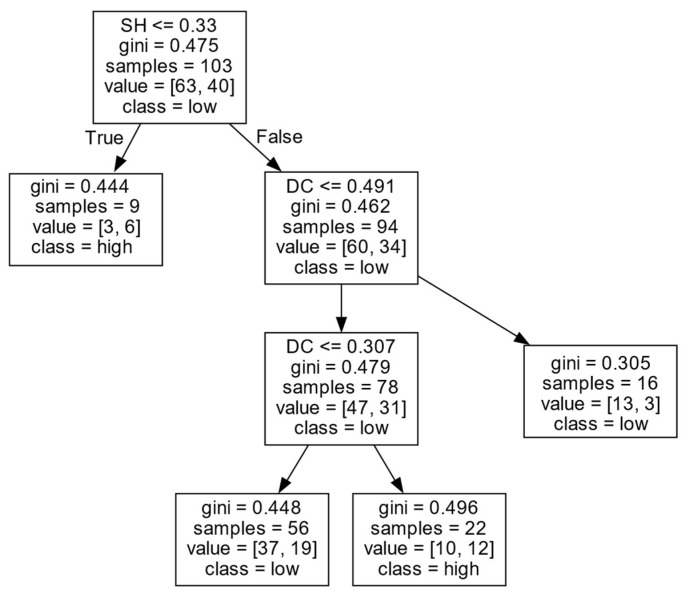
*ERIP* in Cluster I.

As shown in Figure 6 and Table 2, firms’ *ERIP* in Cluster I is mainly influenced by *SH* and *DC*. When the number of *SHs* of a firm is low (SH≤0.330), the firm is more likely to achieve a high *ERIP*. This may be because, if firms have fewer structural holes, there will be more trust among firms and external organizations, thus making inter-organizational collaboration more effective and ultimately conducive to a high *ERIP*.

However, when a firm has a large number of *SHs* (SH<0.330), the *DC* has an inverted U-shaped effect on the firm’s *ERIP*. Specifically, when the *DC* is high (DC>0.491), it is difficult for firms to obtain a high *ERIP*. However, when the *DC* is at a medium level (0.307<DC≤0.491), firms are likely to obtain a high *ERIP*. When the *DC* is at a low level (DC≤0.307), it is difficult for firms to achieve a high *ERIP*. It is possible that, if a firm has more *SHs*, the heterogeneous resources it is exposed to will increase. In this case, if a firm has too many partners, it may have the problem of heterogeneous information overload, resulting in internal coordination difficulties or the need to invest more resources to maintain contact with numerous external partners. If firms have a large number of *SHs* and only have a few external partners, it may lead to opportunistic practices in collaboration and reduce the trust among partners, affecting the efficiency of collaboration innovation. When firms have a large number of *SHs* and a moderate number of partners, they can contact and obtain a great deal of heterogeneous knowledge, which is conducive to integrating heterogeneous knowledge and coordinating external relations, thus helping firms improve their *ERIP*.

(2) Decision rules for *ERIP* in Cluster II.

**Figure 7:**
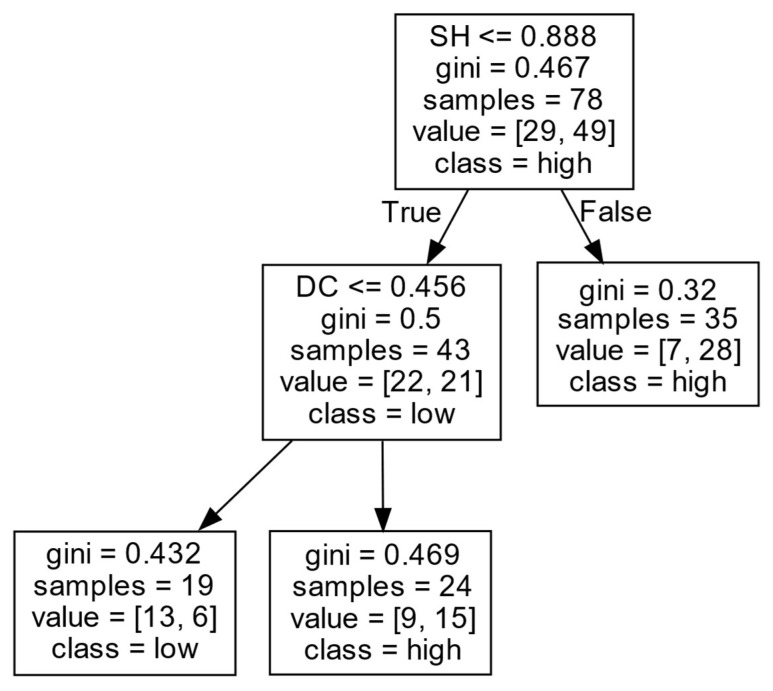
*ERIP* in Cluster II.

According to Figure 7 and Table 3, these firms’ *ERIP* is mainly influenced by the *SH* and *DC*. When there are more *SH*s (SH>0.888), it is easier for these firms to obtain a high *ERIP*. When a firm has a small number of SHs (SH≤0.888), the DC positively affects the firm’s *ERIP*. That is, when the level of *DC* of a firm is high (DC>0.456), the firm is more likely to achieve a high *ERIP*. However, when the *DC* of a firm is at a low level (DC≤0.456), it is difficult for a firm to obtain a high *ERIP*. It is possible that, if the numbers of SHs and external partners are low, the firm may not be in a central position in the collaboration network. As a result, obtaining abundant innovation resources from outside can be difficult, limiting the firm from obtaining a high *ERIP*. However, when the number of external partners is large, firms can gain a large amount of non-redundant heterogeneous knowledge, and new technologies in the industry through inter-organizational interaction, to some extent, make up for the impact of the low number of *SHs* of a firms. Thus, a large number of external partners enable firms to access valuable resources and enhance firms’ *ERIP*.

(3) Decision rules for *ERIP* in Cluster III.

**Figure 8:**
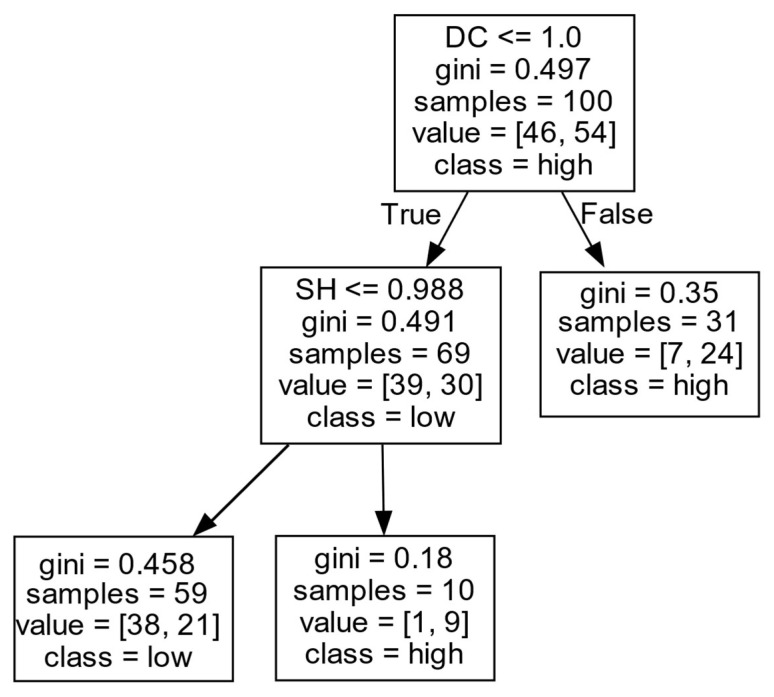
*ERIP* in Cluster III.

As shown in Figure 8 and Table 4, firms’ *ERIP* in Cluster III is mainly influenced by the *DC* and *SH*. When the *DC* of a firm is at a high level (DC>1.000), the firm is likely to achieve a high *ERIP*. When the *DC* of a firm is at a low level (DC≤1.000), the *SH* positively affects the firm’s *ERIP*. Specifically, when the firm has a large number of *SHs* (SH>0.988), the confidence degree of the firms’ high *ERIP* is up to 90%. However, when the number of *SHs* is low (SH≤0.988), it is difficult to obtain a high *ERIP*. This may be because, when the *DC* exceeds a certain threshold, the firm can have more opportunities to exchange information from external partners to improve *ERIP*. Moreover, when the firm has few external partners, it may not be in a favorable central position in the collaboration network. In this case, having more *SHs* can regulate and control the information flow between two unconnected firms, and the firm can obtain more diversified information and resource advantages in the position of an *SH*, which is conducive to improving the *ERIP*.

The decision rules presented in Table 2, Table 3 and Table 4 reveal the following: (1) Two collaboration network structural characteristics (*DC* and *SH*) affect the different firms’ *ERIP* in combination. However, there are no *CC* and *LCC* in any decision rules, indicating that they are not critical factors affecting firms’ *ERIP*. (2) In different firm clusters, the same combination of *DC* and *SH* clearly affects firms’ exploratory innovation performance differently, which verifies the necessity of dividing firms into different clusters to some extent. (3) The average confidence degree reaches up to 71.1%, and some reach 90%, which fully demonstrates that decision rules obtained from the CART algorithm are highly explanatory.

### 6.2. Decision Rules for EIIP

The analysis of the decision rules for *EIIP* in three different firm clusters was undertaken separately as follows.

(1) Decision rules for *EIIP* in Cluster I.

**Figure 9:**
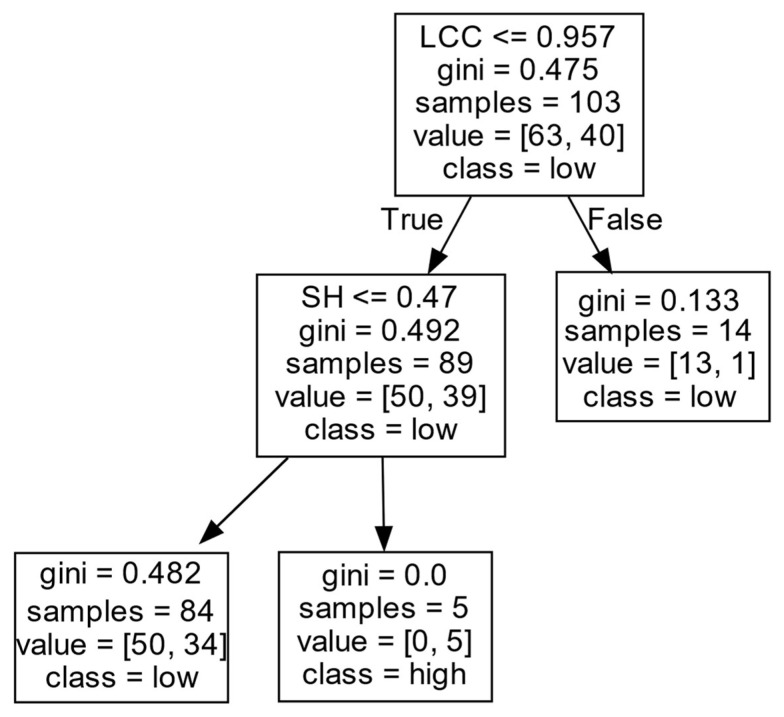
*EIIP* in Cluster I.

As shown in Figure 9 and Table 5, firms’ *EIIP* is mainly affected by the *LCC* and *SH*. When the *LCC* of a firm is at a high level (LCC>0.957), the confidence degree of a firm to obtain a low *EIIP* reaches up to 92.9%. This may be because, when the *LCC* of a firm exceeds a threshold, it will speed up the diffusion of homogeneous information in the network but inhibit the firm in carrying out innovation activities.

When the level of an *LCC* of a firm is low (LCC≤0.957), the *SH* positively affects the firm’s *EIIP*. Specifically, when the number of *SHs* is large (SH>0.470), the confidence degree of the firm in obtaining a high *EIIP* is 100%. However, when the firm has few *SHs* (SH≤0.470), it is difficult for the firm to obtain a high *EIIP*. The reason for this may be that firms with a large number of *SHs* can benefit from non-redundant and heterogeneous knowledge that may not be available in their local network, which can help them optimize their technology and promote the improvement in firms’ *EIIP*. However, if firms have only a few *SHs*, obtaining abundant information from the outside and mastering the initiative of information may be difficult, which limits firms’ access to high *EIIP*.

(2) Decision rules for *EIIP* in Cluster II.

**Figure 10:**
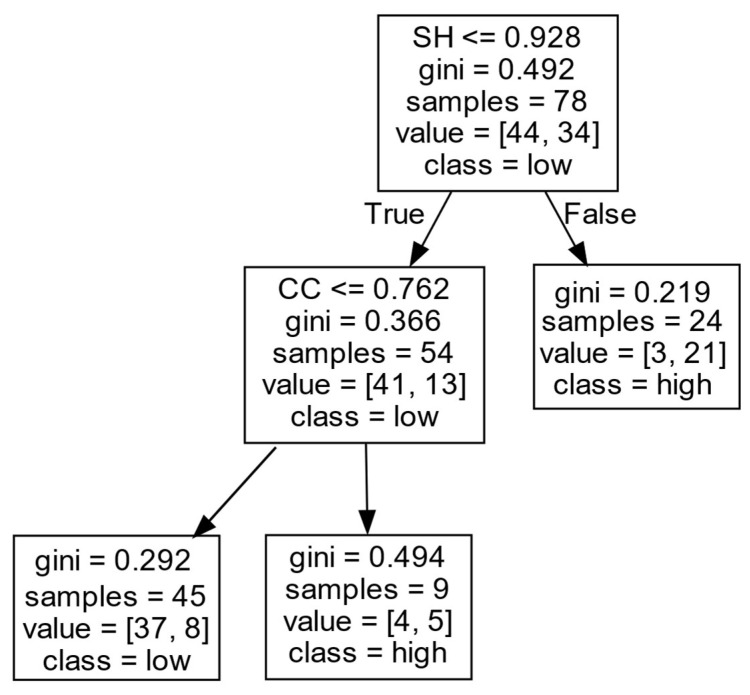
*EIIP* in Cluster II.

According to Figure 10 and Table 6, firms’ *EIIP* in Cluster II is mainly influenced by the *SH* and *CC*. When a firm has a large number of *SHs* (SH>0.928), it is likely to achieve a high *EIIP*. When the firm has only a few *SHs* (SH≤0.928), the *CC* positively affects the firm’s *EIIP*. That is, when the *CC* of a firm is at a high level (CC>0.762), the firm is more likely to obtain a high *EIIP*; otherwise, it will obtain a low *EIIP*.

This could be because the firms with *SHs* have information resources and a control advantage that are conducive to promoting original knowledge and technology and further promoting firms’ *EIIP*. However, when firms have few *SHs*, and the distance between firms and external partners is shorter, the efficiency in absorbing knowledge from collaboration networks can be high. In this case, firms can still absorb a large number of heterogeneous information resources through the convenience of knowledge acquisition, in order to promote the output of *EIIP*. However, when a firm does not have enough structural holes, and the distance required to contact external partners is long, the communication between partners may not be frequent and sufficient, and the non-redundant and heterogeneous knowledge resources obtained by the firm from the outside are less, thus limiting the firm in obtaining a high *EIIP*.

(3) Decision rules for *EIIP* in Cluster III.

**Figure 11:**
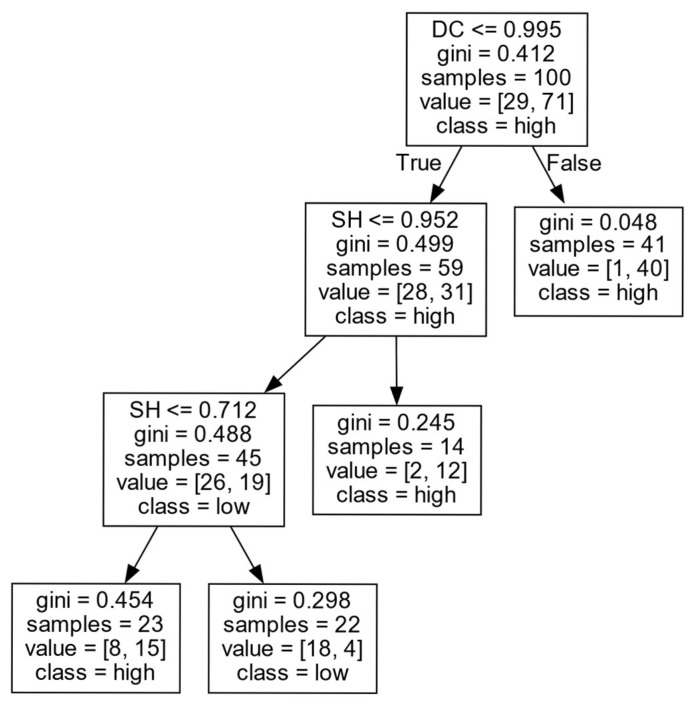
*EIIP* in Cluster III.

As shown in Figure 11 and Table 7, firms’ *EIIP* is mainly influenced by the *DC* and *SH*. When the *DC* of a firm is at a high level (DC>0.995), the confidence of firms in obtaining a high *EIIP* is up to 97.6%. This may be because, when firms have more external partners, they have easy access to key information and knowledge resources, which can help firms maximize the use of existing knowledge to improve the current technology or production mode in order to further improve their *EIIP*.

When the level of *DC* is low (DC≤0.995), the impact of the *SH* on the firms’ *EIIP* is of a “U” type. In other words, when the number of *SHs* is low (SH≤0.712), firms are more likely to obtain high *EIIP*; when the firm has a moderate number of *SHs* (0.712<SH≤0.952), it is difficult for firms to obtain a high *EIIP*. However, when the firm has a large number of *SHs* (SH>0.952), the firm is likely to obtain a high *EIIP*. The following are some possible explanations. When the number of external partners and the number of *SHs* are small, there will be more trust among organizations. With fewer external partners, firms can concentrate on in-depth collaboration with a few key partners, which is conducive to integrating existing knowledge and professional skills, thus enhancing firms’ *EIIP*. However, with the increasing number of *SHs*, the trust among partners may be damaged due to excessive “bridging” behavior, thus affecting collaboration efficiency. Meanwhile, firms may encounter too much heterogeneous knowledge without taking advantage of information control, resulting in heterogeneous information overload and inhibiting exploitative innovation activities. When a firm has a sufficient number of *SHs*, it can make full use of its advantages in information control, grasp the rich combination opportunities among the mastered diversified knowledge elements, and establish new connections among the existing knowledge elements through the in-depth collaboration with existing partners, in order to further improve *EIIP*.

As evidenced by the integrated decision rules in Table 5, Table 6 and Table 7, the following were determined. (1) Four structural characteristics of a collaboration network (*DC*, *CC*, *LCC*, and *SH*) affect firms’ *EIIP* in combination in different clusters. (2) In different clusters, different characteristics or their combination affect firms’ *EIIP*, which to some extent verifies the necessity of dividing firms into different clusters. (3) The average confidence degree reaches 80.8%, and the highest confidence degree reaches is up to 100%, which indicates that decision rules of *EIIP* are highly explanatory.

## 7. Conclusions and Discussion

### 7.1. Conclusions

This study selected the issued patents of 281 firms in the artificial intelligence industry in China as the sample data, and further analyzed the complex nonlinear relationship between the structural characteristics of the inter-organizational collaboration network and firms’ exploratory and exploitative innovation performance. The hierarchical clustering algorithm was used to classify the firms into three clusters, and the CART algorithm was employed to explore the key influencing factors on the exploratory and exploitative innovation performance. The detailed conclusions are as follows.

The firms could be divided into three types of clusters according to the structural characteristics of the collaboration network. There were complex nonlinear relationships between the multi-structural characteristics of the collaboration network and firms’ exploratory and exploitative innovation performance. The four characteristics all impact firms’ innovation performance, in which the *SH* has the most significant impact, while the *CC* and *LCC* have relatively less impact.

For different firm clusters, there were different combinations of structural characteristics of the collaboration network that affect firms’ exploratory and exploitative innovation performance. In different clusters, even the same combination of characteristics can influence firms’ performance differently. This reflects the necessity of dividing firms into clusters to explore the influencing factors and mechanisms of firms’ innovation performance. Therefore, firms should fully consider their own characteristics to optimize their positions in the external collaboration network and further improve innovation performance through effective external collaboration strategies.

The structural characteristics of the collaboration network that affect firms’ exploratory innovation performance differ from those that affect firms’ exploitative innovation performance. The *SH* and *DC* have an essential effect on firms’ exploratory innovation performance in combination, while the *CC* and *LCC* do not affect it. All four characteristics impact firms’ exploitative innovation performance and for different clusters in different combinations. Among them, the *SH* affects all clusters. Therefore, firms should effectively integrate external collaboration resources following their development goals, such as improving exploratory or exploitative innovation performance and obtaining high innovation performance with differentiated development paths.

### 7.2. Theoretical Contributions

Through in-depth analysis and systematic research on the mechanism of the collaboration network affecting firms’ *ERIP* and *EIIP*, three theoretical contributions are as follows. First of all, the application of machine learning methods, such as hierarchical clustering and CART algorithms, in business management is an innovative exploration, which can objectively analyze the complex nonlinear relationship between multiple characteristics of firms and their exploratory and exploitative innovation performance, further mining deeply into potential knowledge and the rules for improving firms’ innovation performance. Second, firms are divided into different clusters according to their characteristics and different types of firms by category, leading to targeted research conclusions and differentiated management strategies. To a certain extent, this breaks the practice via which previous relevant studies rarely used the perspective of differentiation to analyze specific types of firms, and thus further refines and expands the relevant research on important influence factors on firms’ innovation performance. Finally, this study enriches and supplements the research on the complex impact mechanisms between multiple factors and firms’ innovation performance under different management situations. It is also a useful exploration of the research paradigm of management to some extent.

### 7.3. Managerial Implications

The results of this study offer some inspiration for improving firms’ technological innovation development and offer policy implications for governmental agencies in formulating industrial development plans and implementing innovation-driven policies.

Firms should emphasize optimization of the external collaboration network structure to improve innovation performance. Different firms have unique collaboration network structural characteristics and performance improvement mechanisms. To enhance innovation, they must assess their conditions objectively and define clear development goals.

For firms with similar characteristics, the impact factors improving firms’ *ERIP* and *EIIP* may not be unique. Once firms have confirmed their firm type, they can combine the development goals with appropriate resources to implement an optimal development strategy suitable for their needs. Finally, firms can achieve the goal of improving the *ERIP* and *EIIP* by “achieving the same goal through different routes”.

Government departments should pay attention to formulating differentiated strategies and plans for different types of firms and avoid using “one size fits all” standards or models to evaluate, guide, and motivate firms when formulating industrial planning and issuing corresponding policies and measures. Based on comprehensive research, government departments should conduct diversified management and guidance for different types of firms to provide better support. They should give full play to the policy “baton” role to better encourage firms to formulate appropriate development goals and implementation plans based on their reality, and ultimately promote innovation-driven development.

### 7.4. Limitations and Future Research

While this study has significant theoretical contributions and managerial implications, it also suffers from some limitations, which should be addressed in future research. Firstly, only patents from China’s artificial intelligence industry issued from 2017 to 2022 are adopted, and the selected research objects are limited to 281 firms in the same industry. Future research can further expand the sample size, such as by introducing a large number of sample patents across industries, countries, and regions. Secondly, this study only discusses the influence mechanism of firms’ exploratory and exploitative innovation performance from the perspective of the collaboration network. However, factors affecting firms’ innovation performance may include other important factors, such as R&D intensity, organizational redundancy, and human resources, etc. Future research can further broaden the research perspective to analyze and discuss the influence mechanism of other important internal and external factors on firms’ exploratory and exploitative innovation performance, so as to enrich the relevant research. Additionally, this study relied exclusively on patent data to measure the collaboration network and firms’ exploratory and exploitative innovation performance. While patent collaboration is only one of the important forms via which firms can carry out external collaboration, its explanatory scope may be inherently limited. Innovation performance could be further captured through multidimensional variables, such as the revenue from new product sales revenue and the financial performance of firms. Future research could extend the scope by choosing more diversified variables and constructing multi-source heterogeneous datasets, which may enhance both the robustness and interpretability of the research conclusions.

## Figures and Tables

**Table 1:** Corresponding characteristics in different firm clusters.

Cluster	Number	DC	CC	LCC	SH	Proportion of *ERIP* (%)		Proportion of *EIIP* (%)	
I	103	5.466	25.621	0.020	1.012	H	38.8	H	38.8
						L	61.2	L	61.2
II	78	26.128	244.285	0.139	1.489	H	62.8	H	43.6
						L	37.2	L	56.4
III	100	24.160	36.523	0.012	1.342	H	54.0	H	71.0
						L	46.0	L	29.0

**Table 2:** *ERIP* in Cluster I.

Cluster	DC	CC	LCC	SH	Decision Results	Support Degree (%)	Confidence Degree (%)
I	>0.491	-	-	>0.330	low	15.5	81.3
	(0.307, 0.491]	-	-	>0.330	high	21.4	54.5
	≤0.307	-	-	>0.330	low	54.4	66.1
	-	-	-	≤0.330	high	8.7	66.7

**Table 3:** *ERIP* in Cluster II.

Cluster	DC	CC	LCC	SH	Decision Results	Support Degree (%)	Confidence Degree (%)
II	-	-	-	>0.888	high	44.9	80
	>0.456	-	-	≤0.888	high	30.7	62.5
	≤0.456	-	-	≤0.888	low	24.4	68.4

**Table 4:** *ERIP* in Cluster III.

Cluster	DC	CC	LCC	SH	Decision Results	Support Degree (%)	Confidence Degree (%)
III	>1.000	-	-	-	high	31	77.4
	≤1.000	-	-	>0.988	high	10	90
	≤1.000	-	-	≤0.988	low	59	64.4

**Table 5:** *EIIP* in Cluster I.

Cluster	DC	CC	LCC	SH	Decision Results	Support Degree (%)	Confidence Degree (%)
I	-	-	>0.957	-	low	13.6	92.9
	-	-	≤0.957	>0.470	high	4.9	100
	-	-	≤0.957	≤0.470	low	81.6	59.5

**Table 6:** *EIIP* in Cluster II.

Cluster	DC	CC	LCC	SH	Decision Results	Support Degree (%)	Confidence Degree (%)
II	-	-	-	>0.928	high	30.7	87.5
	-	>0.762	-	≤0.928	high	11.5	55.6
	-	≤0.762	-	≤0.928	low	57.7	82.2

**Table 7:** *EIIP* in Cluster III.

Cluster	DC	CC	LCC	SH	Decision Results	Support Degree (%)	Confidence Degree (%)
III	>0.995	-	-	-	high	41	97.6
	≤0.995	-	-	>0.952	high	14	85.7
	≤0.995	-	-	(0.712, 0.952]	low	22	81.8
	≤0.995	-	-	≤0.712	high	23	65.2

## Data Availability

The original contributions presented in this study are included in the supplementary material. Further inquiries can be directed to the first author.

## Supplementary Materials

The following supporting information can be downloaded at: https://www.mdpi.com/article/10.3390/e27060577/s1. Data of focal firms.
