# Supplementary material for: Exploratory and Exploitative Innovation Performance in the Artificial Intelligence Industry in China from the Perspective of a Collaboration Network: A Data-Driven Analysis
Source: Entropy (Basel). 2025 May 29;27(6):577. doi: 10.3390/e27060577 (PMC12191727; doi:10.3390/e27060577)
Supplement: Supplementary file 1 [file entropy-27-00577-s001.zip › entropy-3603759-supplementary.pdf]

**Supplementary Materials:** data_of_focal_firms

| Patentee | Exploratory _Innovation | Exploitative _Innovation | DC | BC | CC | LCC | SH | Cluster |
|---|---|---|---|---|---|---|---|---|
| International Security Technology(Shenzhen) Limited | 3.5 | 1 | 0.35056537952743944 | 0.4278695327024636 | 0.4212941778723078 | 0.9956524920387434 | 0.18659807662198596 | 1 |
| Anhui Jiyuan Software Co., Ltd. | 7.74285714285714 | 0.2 | 0.42887149458057516 | 0.4278695327024636 | 0.9658230337701966 | 0.9999999999982946 | 0.5368983498393014 | 2 |
| Peking University Founder Group Co., Ltd. | 9 | 27.3333333333333 | 0.35056537952743944 | 0.5000076275561611 | 0.4359483642825206 | 1 | 0.5192497718367123 | 1 |
| Beijing Founder Electronics Co., Ltd. | 3.16666666666667 | 36.3333333333333 | 1 | 0.9999993785280666 | 0.438203118958126 | 0.4634827080346329 | 0.49939041145688995 | 3 |
| Beijing Dajia Internet Information Technology Co., Ltd. | 53 | 187.5 | 0.18241484628562907 | 0.4278695327024636 | 0.41833149742681786 | 0.4219990143068912 | 0.36184685290540625 | 1 |
| Beijing Founder Apabi Technology Limited | 0 | 1.5 | 0.7502368209118209 | 0.4278695327024636 | 0.43227589678616174 | 0.5037075637825447 | 0.4740592297877659 | 3 |
| China Techenergy Co., Ltd. | 0 | 1.5 | 0.06800820290420181 | 0.4278695327024636 | 0.5940130759440387 | 0.4219990143068912 | 0.36184685290540625 | 1 |
| Beijing China-Power Information Technology Co., Ltd. | 5.03333333333333 | 1.66666666666667 | 0.06800820290420181 | 1 | 0.9653478781347133 | 0.4219990143068912 | 0.8598686367957954 | 3 |
| Beijing Aerospace Keyi Technology Co., Ltd. | 1 | 0 | 0.18241484628562907 | 0.4278695327024636 | 0.41833149742681786 | 0.4219990143068912 | 0.36184685290540625 | 1 |
| Beijing Kingsoft Office Software, Inc. | 1 | 8.16666666666667 | 0.999600056775284 | 0.4278695327024636 | 0.4212941778723078 | 0.4711833197143965 | 0.232103636356506 | 3 |
| Beijing Boe Optoelectronics Technology Co., Ltd. | 9 | 46.5 | 1 | 0.4278695327024636 | 0.4408666438066607 | 0.4219990143068912 | 0.3618468529054062 | 3 |
| Boe Health Technology Co., Ltd. | 0 | 0.5 | 0.18241484628562907 | 0.4278695327024636 | 0.4408666438066607 | 0.4219990143068912 | 0.36184685290540625 | 1 |
| Beijing Boe Display Technology Co., Ltd. | 6.5 | 8.5 | 0.48271170302490723 | 0.4278695327024636 | 0.4408666438066607 | 0.4219990143068912 | 0.36184685290540625 | 1 |

| | | | | | | | | |
|---|---|---|---|---|---|---|---|---|
| Beijing Jingdong Shangke Information Technology Co., Ltd. | 15.5 | 83 | 1 | 0.4998751922541408 | 0.4212941778723078 | 0.4219990143068912 | 0.40187288895147355 | 3 |
| Beijing Jingdong Century Trading Co., Ltd. | 18.5 | 92.5 | 1 | 0.4278695327024636 | 0.4198177004592919 | 0.4219990143068912 | 0.36184685290540625 | 3 |
| Beijing Kuaishou Technology Co., Ltd. | 1.5 | 1 | 0.18241484628562907 | 0.4278695327024636 | 0.41833149742681786 | 0.4219990143068912 | 0.36184685290540625 | 1 |
| Beijing Kuangshi Technology Co., Ltd. | 45.6666666666667 | 217 | 1 | 0.4278695327024636 | 0.4212941778723078 | 0.4276774813375781 | 0.4093320198563044 | 3 |
| Megvii (Beijing) Technology Co., Ltd. | 42.5 | 96.3333333333333 | 1 | 0.4278695327024636 | 0.4212941778723078 | 0.4276774813375781 | 0.4093320198563044 | 3 |
| Beijing Noitom Technology Ltd. | 9.66666666666667 | 11 | 0.26364124754974233 | 0.4278695327024636 | 0.41833149742681786 | 0.4219990143068912 | 0.36184685290540625 | 1 |
| Beijing Pins Medical Co., Ltd. | 2 | 5 | 0.26364124754974233 | 0.4278695327024636 | 0.8675364530256503 | 0.4219990143068912 | 0.36184685290540625 | 1 |
| Beijing Qihu Technology Co., Ltd. | 13 | 104.5 | 0.26364124754974233 | 0.5617606422815072 | 0.4227607901018912 | 0.4219990143068912 | 0.6173125877472777 | 3 |
| Beijing Automotive Group Co., Ltd. | 2.5 | 1.5 | 0.5237701734645818 | 0.4278695327024636 | 0.41833149742681786 | 0.4219990143068912 | 0.36184685290540625 | 1 |
| Beijing Automotive Technology Center | 5 | 1.5 | 0.5237701734645818 | 0.4278695327024636 | 0.41833149742681786 | 0.4219990143068912 | 0.36184685290540625 | 1 |
| Beijing Sankuai Online Technology Co., Ltd. | 51 | 236.5 | 0.18241484628562907 | 0.7116893099893431 | 0.42421739815171033 | 0.4219990143068912 | 0.9550299900644101 | 3 |
| Beijing Samsung Telecom R&D Center | 1.5 | 8.5 | 0.9998710574239954 | 0.4278695327024636 | 0.42421739815171033 | 0.4219990143068912 | 0.36184685290540625 | 3 |
| Beijing Roborock Technology Co., Ltd. | 3 | 4 | 0.35056537952743944 | 0.999974158421422 | 0.4341219910300546 | 0.4219990143068912 | 0.793097574254146 | 3 |
| Beijing Sifang Automation Co., Ltd. | 3 | 1.83333333333333 | 0.42887149458057516 | 0.7900224960492248 | 0.4271000486954222 | 0.9999899351919338 | 0.6402995819471364 | 1 |
| Beijing Xiaomi Mobile Software Co., Ltd. | 59.5 | 549 | 0.8929264803996686 | 1 | 0.44130602330709073 | 0.4219990143068912 | 0.9970609701851969 | 3 |

| | | | | | | | | |
|---|---|---|---|---|---|---|---|---|
| Beijing Eyecool Technology Co., Ltd. | 9.83333333333333 | 19.1666666666667 | 1 | 0.5337393336815657 | 0.4304103986976749 | 0.4571800811799145 | 0.5000015688968944 | 3 |
| Beijing Techshino Technology Co., Ltd. | 2.83333333333333 | 12.1666666666667 | 1 | 0.9999999997546419 | 0.4327392518864463 | 0.4585706870261263 | 0.493263511018143 | 3 |
| Beijing Smartmi Technology Co., Ltd. | 1 | 0.5 | 0.42887149458057516 | 0.9999999542638712 | 0.4341219910300546 | 0.4219990143068912 | 0.7431828698121301 | 3 |
| Beijing Zhongdian Puhua Information Technology Co., Ltd. | 2 | 3.05952380952381 | 0.9381324887223157 | 0.9999999999238226 | 0.9756694172921171 | 0.9999999999999999 | 0.952998070463961 | 2 |
| Beijing Zhongdun Security Technology Limited | 1 | 0.5 | 0.11612065989230397 | 0.4278695327024636 | 0.4198177004592919 | 0.4219990143068912 | 0.36184685290540625 | 1 |
| Airwing Aviation Technology Limited | 0 | 0.333333333333333 | 0.48271170302490723 | 0.48133505508035057 | 0.9661368490503738 | 0.9999996120100095 | 0.501033131119926 | 2 |
| Beijing Zongheng Electro-Mechanical Technology Development Co., Ltd. | 0.583333333333333 | 0 | 0.42887149458057516 | 1 | 0.4454141062447515 | 0.9999899351919338 | 0.8452155442761276 | 3 |
| Bgrimm Machinery and Automation Technology Co., Ltd. | 2 | 0.5 | 0.11612065989230397 | 0.4998751922541408 | 0.4212941778723078 | 0.4219990143068912 | 0.793097574254146 | 3 |
| Beijing Sanlian Yonghui Technology Co., Ltd. | 0.5 | 2 | 0.26364124754974233 | 0.4278695327024636 | 0.8675364530256503 | 0.4219990143068912 | 0.36184685290540625 | 1 |
| Chengdu Boe Optoelectronics Technology Co., Ltd. | 13 | 14 | 0.9381324887223157 | 0.4278695327024636 | 0.4408666438066607 | 0.4219990143068912 | 0.36184685290540625 | 3 |
| Chengdu Image Design Technology Co., Ltd. | 0.5 | 0 | 0.48271170302490723 | 0.4998751922541408 | 0.4212941778723078 | 0.4219990143068912 | 0.8598686367957954 | 3 |
| Daya Bay Nuclear Power Operations And Management Co., Ltd. | 0.652380952380952 | 0 | 0.7502368209118209 | 0.4362086563810849 | 0.6003343407233682 | 0.9999999683001379 | 0.6460048926895773 | 3 |
| Dongguan Solution 33 Electronic Technology Co., Ltd. | 0.5 | 1.75 | 0.663368062786774 | 0.4278695327024636 | 0.4271000486954222 | 0.9999704258833632 | 0.5052687360991258 | 1 |
| Dongguan Yulong Telecommunications Scientific Co., Ltd. | 1 | 0 | 0.06800820290420181 | 0.4278695327024636 | 0.41833149742681786 | 0.4219990143068912 | 0.36184685290540625 | 1 |
| Neusoft Medical Systems Co., Ltd. | 17.5 | 24 | 0.06800820290420181 | 0.4278695327024636 | 0.41833149742681786 | 0.4219990143068912 | 0.36184685290540625 | 1 |

| | | | | | | | | |
|---|---|---|---|---|---|---|---|---|
| Ordos Yuansheng Optoelectronics Co., Ltd. | 1 | 2 | 0.26364124754974233 | 0.4278695327024636 | 0.4408666438066607 | 0.4219990143068912 | 0.36184685290540625 | 1 |
| Pan Asia Technical Automotive Center Company Limited. | 1 | 1.5 | 0.18241484628562907 | 0.5617606422815072 | 0.4227607901018912 | 0.4219990143068912 | 0.8598686367957954 | 3 |
| Fiberhome Telecommunication Technologies Co., Ltd. | 21 | 31.5 | 0.11612065989230397 | 0.4998751922541408 | 0.4212941778723078 | 0.4219990143068912 | 0.793097574254146 | 3 |
| Foshan Shunde Midea Electrical Heating Appliances Manufacturing Company Limited | 13 | 8 | 0.11612065989230397 | 0.4278695327024636 | 0.43227589678616174 | 0.4219990143068912 | 0.36184685290540625 | 1 |
| First Dome Corp Telecom Co., Ltd | 4.5 | 2 | 0.35056537952743944 | 0.4278695327024636 | 0.41833149742681786 | 0.4219990143068912 | 0.36184685290540625 | 1 |
| Futaihua Industrial (Shenzhen) Co., Ltd. | 3.5 | 4 | 0.9381324887223157 | 0.4278695327024636 | 0.6304923025609243 | 0.4219990143068912 | 0.36184685290540625 | 3 |
| K-Tronics (Suzhou) Technology Co., Ltd. | 0 | 1.5 | 0.18241484628562907 | 0.4278695327024636 | 0.4408666438066607 | 0.4219990143068912 | 0.36184685290540625 | 1 |
| Lite-On Electronics (Guangzhou) Co., Ltd. | 1.5 | 1.5 | 0.11612065989230397 | 0.4278695327024636 | 0.4198177004592919 | 0.4219990143068912 | 0.36184685290540625 | 1 |
| LITE-ON Technology Corporation | 2 | 7 | 0.18241484628562907 | 0.4998751922541408 | 0.4212941778723078 | 0.4219990143068912 | 0.6952794734091069 | 3 |
| Guangdong Power Grid Corporation | 65.5 | 210.833333333333 | 1 | 1 | 0.8821448924641054 | 0.43273274281699536 | 0.9989843892410877 | 3 |
| Guangdong Electric Power Science Academe | 9.5 | 23.5 | 0.5825689895709085 | 1 | 0.8847199207205885 | 0.4219990143068912 | 0.9979798917594225 | 3 |
| Guangdong Power Grid Co., Ltd. Dongguan Power Supply Bureau | 16 | 6.5 | 0.5825689895709085 | 1 | 0.7295751743060545 | 0.4219990143068912 | 0.4999692648862635 | 1 |
| Guangdong Power Grid Co., Ltd. Foshan Power Supply Bureau | 34 | 0 | 0.18241484628562907 | 0.4278695327024636 | 0.7271874666257564 | 0.4219990143068912 | 0.36184685290540625 | 1 |
| Guangdong Power Grid Co., Ltd. Huizhou Power Supply Bureau | 10 | 1 | 0.48271170302490723 | 0.4278695327024636 | 0.7271874666257564 | 0.4219990143068912 | 0.36184685290540625 | 1 |
| Guangdong Power Grid Co., Ltd. Zhongshan Power Supply Bureau | 15.5 | 4.5 | 0.26364124754974233 | 0.4278695327024636 | 0.7271874666257564 | 0.4219990143068912 | 0.36184685290540625 | 1 |

| | | | | | | | | |
|---|---|---|---|---|---|---|---|---|
| Guangdong Midea White Appliance Technology Innovation Center Co., Ltd. | 4 | 2.5 | 0.35056537952743944 | 0.4278695327024636 | 0.43227589678616174 | 0.4219990143068912 | 0.36184685290540625 | 1 |
| Guangdong Midea Microwave And Electrical Appliances Manufacturing Co., Ltd. | 2.5 | 3.5 | 0.26364124754974233 | 0.9999950421389212 | 0.4341219910300546 | 0.4219990143068912 | 0.6173125877472777 | 3 |
| Gd Midea Environment Appliances Mfg. Co., Ltd. | 0.5 | 0.5 | 0.18241484628562907 | 0.4278695327024636 | 0.43227589678616174 | 0.4219990143068912 | 0.36184685290540625 | 1 |
| Gd Midea Air-Conditioning Equipment Co., Ltd. | 5 | 27.5 | 0.9999999998859662 | 0.4278695327024636 | 0.43227589678616174 | 0.4219990143068912 | 0.36184685290540625 | 3 |
| Guangxi Power Grid Co., Ltd. Electric Power Research Institute | 12 | 24.7 | 0.18241484628562907 | 1 | 0.8735893297502693 | 0.4219990143068912 | 0.9899291849526439 | 3 |
| Guangzhou Power Supply Co., Ltd. | 2.5 | 0.5 | 0.06800820290420181 | 1 | 0.6593633120650443 | 0.4219990143068912 | 0.8598686367957954 | 3 |
| Guangzhou Kingsoft Mobile Technology Co., Ltd. | 1 | 4.66666666666667 | 0.9970035308028009 | 0.4278695327024636 | 0.4212941778723078 | 0.47895913375766697 | 0.11077371939029573 | 3 |
| Samsung Guangzhou Mobile R&D Center | 1 | 1.5 | 0.35056537952743944 | 0.4278695327024636 | 0.42421739815171033 | 0.4219990143068912 | 0.36184685290540625 | 1 |
| Guangzhou Shirui Electronics Co., Ltd. | 16.5 | 74 | 1 | 0.4278695327024636 | 0.4227607901018912 | 0.4219990143068912 | 0.3618468529054034 | 3 |
| Guangzhou Shiyuan Electronic Technology Co., Ltd. | 23 | 212.5 | 1 | 0.9853409345508982 | 0.4271000486954222 | 0.4219990143068912 | 0.7665038885869533 | 3 |
| Guangzhou Shizhen Information Technology Co., Ltd. | 2.5 | 11.5 | 0.9999973793726163 | 0.4278695327024636 | 0.4227607901018912 | 0.4219990143068912 | 0.36184685290540625 | 3 |
| Guangzhou Shuangyou Biotechnology Co., Ltd. | 0 | 0.5 | 0.5001370302919633 | 0.4278695327024636 | 0.6476540388425754 | 0.6797196065514214 | 0.46275219173242016 | 1 |
| Guangzhou Xicoo Medical Technology Co., Ltd. | 2.5 | 4 | 0.8295080017104791 | 0.4278695327024636 | 0.4227607901018912 | 0.4219990143068912 | 0.36184685290540625 | 3 |
| Nari Technology Co., Ltd. | 20.8277777777778 | 12.1 | 0.9999999999979519 | 1 | 0.9759485965525965 | 0.9850561449586901 | 0.9737614001122661 | 2 |
| Nari Technology Nanjing Control Systems Co., Ltd. | 12.2944444444444 | 1.33333333333333 | 0.9927619881530132 | 1 | 0.971203955050123 | 0.9999966284363773 | 0.9108220242508275 | 2 |

| | | | | | | | | |
|---|---|---|---|---|---|---|---|---|
| China International Safety Technology Co., Ltd. | 1.5 | 2.22044604925031e-16 | 0.35056537952743944 | 0.4278695327024636 | 0.4212941778723078 | 0.9956524920387434 | 0.18659807662198596 | 1 |
| State Grid Corporation Of China | 39.2873015873016 | 304.305952380952 | 1 | 1 | 0.9999913575923348 | 0.4390086913738723 | 0.9997272094082297 | 2 |
| National Energy Investment Group Co., Ltd. | 1.45 | 0 | 0.42887149458057516 | 1 | 0.4377547018063007 | 0.9999899351919338 | 0.8807061825393782 | 3 |
| State Grid Anhui Electric Power Co., Ltd., Electric Power Research Institute | 8.25 | 1.33333333333333 | 0.663368062786774 | 0.9102010010700512 | 0.9670642400094723 | 0.9991762612596617 | 0.7829652143799142 | 2 |
| State Grid Anhui Electric Power Co., Ltd. Lu'an Power Supply Company | 1.08333333333333 | 0 | 0.42887149458057516 | 0.7517360588132834 | 0.9669111302930278 | 1 | 0.8283065515494102 | 2 |
| State Grid Beijing Electric Power Company | 3.5 | 8.25 | 0.9927619881530132 | 1 | 0.9703590009739574 | 0.9842914939856674 | 0.9566948704708983 | 2 |
| Wuhan Nari Limited Liability Company Of State Grid Electric Power Research Institute | 8.92619047619048 | 1.16666666666667 | 0.35056537952743944 | 1 | 0.9699360235162271 | 1 | 0.9713985137292412 | 2 |
| State Grid Electric Power Research Institute | 7.63730158730159 | 8.88178419700125e-16 | 0.48271170302490723 | 1 | 0.9716121608342978 | 1 | 0.9599315028160902 | 2 |
| State Grid Fujian Electric Power Co., Ltd. | 9.16666666666667 | 22.5 | 1 | 1 | 0.976369812353738 | 0.6485495676641461 | 0.9716835524976658 | 2 |
| State Grid Fujian Electric Power Co., Ltd., Electric Power Research Institute | 5.33333333333333 | 1.61666666666667 | 0.48271170302490723 | 0.4278695327024636 | 0.9690751793416664 | 1 | 0.8612538311943967 | 2 |
| State Grid Fuzhou Power Supply Company | 1.41666666666667 | 0.333333333333333 | 0.9381324887223157 | 0.5060680509291023 | 0.967368724715669 | 0.9839565190908315 | 0.762592396525783 | 2 |
| State Grid Hebei Electric Power Company | 16.7333333333333 | 20.55 | 0.48271170302490723 | 0.4998751922541408 | 0.9661368490503738 | 0.9999996120100095 | 0.6483667130648089 | 2 |
| State Grid Hebei Electric Power Company Electric Power Research Institute | 8.16666666666666 | 1 | 0.5001370302919633 | 0.490254661778110 06 | 0.9662928745720816 | 0.9999998808209744 | 0.7232666053654364 | 2 |
| State Grid Hebei Electric Power Co., Ltd. Maintenance Branch Company | 0.333333333333333 | 0 | 0.03664507312993 | 0.4278695327024636 | 0.9647060009664058 | 0.4219990143068912 | 0.36184685290540625 | 1 |
| State Grid Henan Electric Power Company | 7.28333333333333 | 0.916666666666666 | 0.35056537952743944 | 0.49689309974554635 | 0.9661368490503738 | 1 | 0.7647380956003953 | 2 |

| | | | | | | | | |
|---|---|---|---|---|---|---|---|---|
| Henan Electric Power Company Zhengzhou Power Supply Company | 2.66666666666667 | 0.666666666666667 | 0.35056537952743944 | 0.9560896751423812 | 0.96738838883956263 | 1 | 0.7512497119194091 | 2 |
| State Grid Hubei Electric Power Company Extra-High Voltage Company | 5.66666666666667 | 1.5 | 0.5001370302919633 | 0.5272657700657678 | 0.9662928745720816 | 0.9999998808209744 | 0.7956779451915561 | 2 |
| State Grid Hunan Electric Power Company | 3.58333333333333 | 11.9583333333333 | 0.9999999999999927 | 1 | 0.9701481284909107 | 0.6157794557656971 | 0.8074790206345618 | 2 |
| State Grid Hunan Electric Power Co., Ltd., Electric Power Research Institute | 3.58333333333333 | 1 | 0.5825689895709085 | 0.4278695327024636 | 0.9656652410617198 | 0.9729010024545853 | 0.5421700662202913 | 2 |
| State Grid Hunan Electric Power Co., Ltd. Disaster Prevention and Mitigation Center | 1.25 | 1 | 0.35056537952743944 | 0.4278695327024636 | 0.9651883049675989 | 0.9956524920387434 | 0.5049925769919358 | 2 |
| State Grid Jibei Electric Power Company Extra-High Voltage Company | 0 | 0.833333333333333 | 0.48271170302490723 | 0.4278695327024636 | 0.9651883049675989 | 0.8070293551164697 | 0.5000314410275305 | 2 |
| State Grid Jibei Electric Power Co., Ltd. Electric Power Research Institute | 0.4 | 0.45 | 0.5825689895709085 | 0.8558003448062255 | 0.9687105750790354 | 0.9999999999994097 | 0.8149851615403688 | 2 |
| Information And Communication Branch Of State Grid Jibei Electric Power Co., Ltd. | 0.333333333333333 | 0.416666666666667 | 0.5825689895709085 | 1 | 0.967368724715669 | 0.9999999994041386 | 0.9102173566035465 | 2 |
| Jiangsu Provincial Electric Power Corporation | 26.7666666666667 | 101.899206349206 | 1 | 1 | 0.98964540936538 | 0.49983451793496786 | 0.9755818969502951 | 2 |
| Changzhou Power Supply Branch Of State Grid Jiangsu Electric Power Co., Ltd. | 3.36666666666667 | 0.666666666666666 | 0.5001370302919633 | 0.4278695327024636 | 0.9658230337701966 | 0.9998297941335809 | 0.6337689110248446 | 2 |
| State Grid Jiangsu Electric Power Co., Ltd. Electric Power Research Institute | 7.67619047619048 | 13.3857142857143 | 1 | 1 | 0.9821245540827003 | 0.6150153253538876 | 0.9461207371494088 | 2 |
| Jiangsu Electric Power Company Lianyungang Power Supply Company | 2.47619047619048 | 0 | 0.26364124754974233 | 0.4278695327024636 | 0.9651883049675989 | 0.9999971192244244 | 0.5053431094064536 | 2 |
| Jiangsu Electric Power Company Nanjing Power Supply Company | 7.83333333333333 | 3.31666666666667 | 0.999989562420184 | 1 | 0.9763166689123183 | 0.999312005798607 | 0.9796507917410504 | 2 |
| Jiangsu Electric Power Company Nantong Power Supply Company | 8.5 | 3.33333333333333 | 0.26364124754974233 | 0.4278695327024636 | 0.9658230337701966 | 1 | 0.6615170940334669 | 2 |
| Jiangsu Electric Power Company Suzhou Power Supply Company | 11.7777777777778 | 1 | 0.35056537952743944 | 0.4278695327024636 | 0.9680603185723767 | 1 | 0.8457377135647517 | 2 |

| | | | | | | | | |
|---|---|---|---|---|---|---|---|---|
| Jiangsu Electric Power Company Wuxi Power Supply Company | 6.5 | 0.25 | 0.7502368209118209 | 1 | 0.9680454124672891 | 0.9999999683001379 | 0.8935800709105005 | 2 |
| Jiangsu Electric Power Company Yancheng Power Supply Company | 1.33333333333333 | 1.16666666666667 | 0.8929264803996686 | 0.9999981929783865 | 0.9684163615025624 | 0.9999999980181261 | 0.9263053703873833 | 2 |
| Liaoning Electric Power Company Anshan Power Supply Company | 1 | 0 | 0.42887149458057516 | 0.4887104270443359 | 0.9661368490503738 | 1 | 0.712320778379959 | 2 |
| State Grid Liaoning Electric Power Supply Co., Ltd. | 6.2 | 3.5 | 0.7502368209118209 | 1 | 0.9747875988121067 | 0.9999999995715074 | 0.8947151459472334 | 2 |
| State Grid Qinghai Electric Power Company | 10.6166666666667 | 2.83333333333333 | 0.5825689895709085 | 1 | 0.9753342768422059 | 1 | 0.9894905406071314 | 2 |
| State Grid Shandong Electric Power Company | 25.8333333333333 | 58.5166666666667 | 0.9988586920675357 | 1 | 0.9695797628647526 | 0.999243717083318 | 0.9598583005169147 | 2 |
| State Grid Shandong Electric Power Company Extra-High Voltage Company | 1.5 | 0.5 | 0.26364124754974233 | 0.4278695327024636 | 0.9651883049675989 | 0.9999971192244244 | 0.4978697348102514 | 2 |
| Shandong Electric Power Research Institute | 5.75 | 12 | 0.9999999999839989 | 1 | 0.9710530716415618 | 0.6902219861980974 | 0.9331151837905289 | 2 |
| Shandong Electric Power Company Jinan Power Supply Company | 0.533333333333333 | 0 | 0.18241484628562907 | 0.49200804668338044 | 0.9662928745720816 | 1 | 0.8710647437407856 | 2 |
| State Grid Shanghai Electric Power Company | 17.0833333333333 | 9.95 | 0.9999993946406689 | 1 | 0.9743252504978567 | 0.6915701380146303 | 0.9948423559906114 | 2 |
| State Grid Tianjin Electric Power Company | 8.5 | 9.66666666666667 | 0.48271170302490723 | 0.4278695327024636 | 0.9651883049675989 | 0.8070293551164697 | 0.4959941428246453 | 2 |
| State Grid Xinjiang Electric Power Co., Ltd. Electric Power Research Institute | 0 | 0.5 | 0.11612065989230397 | 1 | 0.9655861227952013 | 0.4219990143068912 | 0.793097574254146 | 3 |
| State Grid Xin Yuan Company Limited | 1.83333333333333 | 0.333333333333333 | 0.06800820290420181 | 0.4278695327024636 | 0.9651883049675989 | 1 | 0.5021304212223745 | 2 |
| State Grid Xintong Yili Technology Co., Ltd. | 3.97619047619048 | 2.10833333333333 | 0.7502368209118209 | 0.5008545493248705 | 0.9682684072700125 | 1 | 0.9188700499762177 | 2 |
| National Network Information And Communication Industry Group Co., Ltd. | 15.8666666666667 | 15.627380952381 | 0.9999999992510294 | 1 | 0.9783890439950094 | 0.9986300776016386 | 0.9848320220497744 | 2 |

| | | | | | | | | |
|---|---|---|---|---|---|---|---|---|
| State Grid Zhejiang Hangzhou Yuhang District Power Supply Company | 2.1166666666 6667 | 0.333333333 333333 | 0.182414846 28562907 | 1 | 0.83027350604 86559 | 1 | 0.884444126 7371249 | 2 |
| State Grid Zhejiang Electric Power Co., Ltd. | 52.55 | 98.19285714 28571 | 1 | 1 | 0.98474395330 15454 | 0.84508888 8429025 | 0.988306057 0756707 | 2 |
| Zhejiang Electric Power Company Hangzhou Power Supply Company | 7.7 | 5.15 | 0.482711703 02490723 | 1 | 0.96911869669 50984 | 0.99999961 20100095 | 0.888288855 1779634 | 2 |
| Zhejiang Electric Power Company Huzhou Power Supply Company | 11.833333333 3333 | 0 | 0.582568989 5709085 | 0.47937350 499153036 | 0.96566524106 17198 | 0.81641670 65804123 | 0.583758115 0723251 | 2 |
| Zhejiang Electric Power Company Lishui Power Supply Company | 0 | 0.5 | 0.263641247 54974233 | 1 | 0.96726241765 39485 | 1 | 0.872929067 7473186 | 2 |
| Zhejiang Electric Power Company Ningbo Power Supply Company | 2.8166666666 6667 | 3.666666666 66667 | 0.983914622 7189915 | 1 | 0.97102556838 29775 | 0.99458982 05095249 | 0.981685910 0608322 | 2 |
| Zhejiang Electric Power Company Shaoxing Power Supply Company | 7.9166666666 6667 | 1.333333333 33333 | 0.350565379 52743944 | 0.49880718 021616083 | 0.96875451276 30086 | 1 | 0.901993176 7323575 | 2 |
| Zhejiang Electric Power Company Taizhou Power Supply Company | 5.3333333333 3333 | 0 | 0.500137030 2919633 | 0.47937350 499153036 | 0.96566524106 17198 | 0.97633618 74728148 | 0.531575098 196196 | 2 |
| Zhejiang Electric Power Company Wenzhou Power Supply Company | 2.8333333333 3333 | 0.5 | 0.500137030 2919633 | 0.42786953 27024636 | 0.96680360835 44164 | 0.99982979 41335809 | 0.693387193 9905069 | 2 |
| State Grid Zhejiang Yuyao Power Supply Co., Ltd. | 0.4 | 0 | 0.263641247 54974233 | 1 | 0.96675744036 6672 | 0.99999711 92244244 | 0.873471776 0468396 | 2 |
| State Grid Smart Grid Research Institute Co., Ltd. | 2.9166666666 6667 | 9.826190476 19048 | 0.999999995 4746681 | 1 | 0.97442466110 0549 | 0.98185474 24528388 | 0.946887344 2489194 | 2 |
| State Grid Chongqing Electric Power Company | 7.0833333333 3333 | 2.666666666 66667 | 0.482711703 02490723 | 0.47937350 499153036 | 0.96566524106 17198 | 0.99812359 05421787 | 0.507018843 9034523 | 2 |
| Beijing Haier Guangke Digital Technology Co., Ltd | 5 | 9.166666666 66667 | 0.482711703 02490723 | 0.42786953 27024636 | 0.42276079010 18912 | 0.80702935 51164697 | 0.477799173 4158749 | 1 |
| Hangzhou Honyar Electrical Co., Ltd. | 0.5 | 2.5 | 0.182414846 28562907 | 0.49987519 22541408 | 0.42129417787 23078 | 0.42199901 43068912 | 0.695279473 4091069 | 3 |
| Hefei Boe Optoelectronics Technology Co., Ltd. | 8.5 | 2.5 | 0.482711703 02490723 | 0.42786953 27024636 | 0.44086664380 66607 | 0.42199901 43068912 | 0.361846852 90540625 | 1 |

| | | | | | | | | |
|---|---|---|---|---|---|---|---|---|
| Hefei Xinsheng Optoelectronics Technology Co., Ltd | 3.5 | 1.5 | 0.35056537952743944 | 0.4278695327024636 | 0.4408666438066607 | 0.4219990143068912 | 0.36184685290540625 | 1 |
| Pegatron Corporation | 9.5 | 17 | 0.26364124754974233 | 0.4998751922541408 | 0.4212941778723078 | 0.4219990143068912 | 0.6173125877472777 | 3 |
| Hongfujin Precision Industry (Shenzhen) Co., Ltd. | 1.5 | 1 | 0.11612065989230397 | 1 | 0.8711558229788038 | 0.4219990143068912 | 0.793097574254146 | 3 |
| Hon Hai Precision Industry Co., Ltd. | 5.5 | 7 | 0.9970035308028009 | 1 | 0.7333518273343309 | 0.4219990143068912 | 0.6519671543000738 | 3 |
| Hunan Great Wall Medical Technology Co., Ltd. | 0.5 | 0 | 0.03664507312993 | 0.4278695327024636 | 0.41833149742681786 | 0.4219990143068912 | 0.36184685290540625 | 1 |
| Hunan Great Wall Galaxy Technology Co., Ltd. | 1.5 | 1.5 | 0.11612065989230397 | 0.4278695327024636 | 0.41833149742681786 | 0.4219990143068912 | 0.36184685290540625 | 1 |
| North China Electric Power Research Institute Co., Ltd. | 0 | 1.1 | 0.8929264803996686 | 1 | 0.96986504711265 | 0.9999899351919338 | 0.8874571549115691 | 2 |
| Huadong Electric Power Experimental and Research Institute Co., Ltd. | 3.33333333333333 | 0.5 | 0.18241484628562907 | 0.4278695327024636 | 0.8373736544259601 | 1 | 0.49922269277682313 | 2 |
| Shenzhen Huawei Technologies Software Co., Ltd | 154 | 717.833333333333 | 0.9999999992510294 | 1 | 0.905332217800835 | 0.44891843002015824 | 0.9711785778386854 | 3 |
| China Communication Technology Co., Ltd. | 1.5 | 1 | 0.26364124754974233 | 0.4278695327024636 | 0.41833149742681786 | 0.4219990143068912 | 0.36184685290540625 | 1 |
| Qisda Corporation | 5.5 | 6 | 0.7502368209118209 | 0.4278695327024636 | 0.4198177004592919 | 0.4219990143068912 | 0.36184685290540625 | 3 |
| Suyuan Group Jiangsu Information Technology Co., Ltd. | 9.5 | 2.33333333333333 | 0.26364124754974233 | 0.9999999999999999 | 0.8748601672727934 | 1 | 0.7924018923895342 | 2 |
| Jiangsu Frontier Electric Power Technology Co., Ltd. | 15.0833333333333 | 17.25 | 0.9970035308028009 | 0.48263150561194473 | 0.9666031687547725 | 0.6556055255875785 | 0.737182852695924 | 2 |
| Jiangsu Power Testing Research Institute Co., Ltd. | 7.56904761904762 | 5.07619047619048 | 0.8929264803996686 | 0.5658177024672602 | 0.9687983998613847 | 0.999243717083318 | 0.82242693887747 | 2 |
| Jiangsu Yuyue Information System Co., Ltd. | 2.2 | 4.11666666666667 | 0.9999999998859662 | 0.4278695327024636 | 0.4271000486954222 | 0.5016880125775625 | 0.5199351024084571 | 3 |

| | | | | | | | | | |
|---|---|---|---|---|---|---|---|---|---|
| Jiangsu Yuyue Medical Equipment And Supply Co., Ltd. | 2.2 | 4.11666666666667 | 0.9999999999979519 | 0.4278695327024636 | 0.4271000486954222 | 0.5000068383457262 | 0.5393853417673535 | 3 |
| Jiangsu Zhengxin Intelligent Technology Co., Ltd. | 1 | 5.5 | 0.5237701734645818 | 0.4278695327024636 | 0.41833149742681786 | 0.4219990143068912 | 0.36184685290540625 | 1 |
| Boe Optical Science And Technology Co., Ltd. | 1 | 0 | 0.11612065989230397 | 0.4278695327024636 | 0.4408666438066607 | 0.4219990143068912 | 0.36184685290540625 | 1 |
| Boe Technology Group Co., Ltd. | 38 | 543.5 | 1 | 1 | 0.46039861034690044 | 0.4219990143068912 | 0.9933104479224556 | 3 |
| Hkust Intelligent Electrical Technology Co., Ltd. | 0.333333333333333 | 0 | 0.5001370302919633 | 0.49392919138324576 | 0.42421739815171033 | 0.9763361874728148 | 0.5694413625203645 | 1 |
| Csg Smart Science and Technology Co., Ltd. | 0.333333333333333 | 0 | 0.42887149458057516 | 0.45697199239856545 | 0.42421739815171033 | 0.9999899351919338 | 0.3590829285959153 | 1 |
| Kostal (Shanghai) Management Co., Ltd. | 1 | 0.5 | 0.11612065989230397 | 0.4278695327024636 | 0.41833149742681786 | 0.4219990143068912 | 0.36184685290540625 | 1 |
| Kunshan New Flat Panel Display Technology Center Co., Ltd. | 0.5 | 0 | 0.11612065989230397 | 0.4278695327024636 | 0.41833149742681786 | 0.4219990143068912 | 0.36184685290540625 | 1 |
| Kunshan Govisionox Optoelectronics Co., Ltd. | 10.5 | 19 | 0.11612065989230397 | 0.4278695327024636 | 0.41833149742681786 | 0.4219990143068912 | 0.36184685290540625 | 1 |
| Nextvpu (Kunshan) Co., Ltd. | 0.333333333333333 | 0 | 0.42887149458057516 | 0.4278695327024636 | 0.4212941778723078 | 0.936819075702284 | 0.3961658527234138 | 1 |
| China Unicom Digital Technology Co., Ltd. | 1 | 0 | 0.11612065989230397 | 0.4278695327024636 | 0.41833149742681786 | 0.4219990143068912 | 0.36184685290540625 | 1 |
| Lenovo (Beijing) Co., Ltd. | 30.5 | 807.25 | 0.11612065989230397 | 1 | 0.64987008683182 | 0.4219990143068912 | 0.793097574254146 | 3 |
| Lingdong Nuclear Power Co., Ltd. | 0.852380952380952 | 0.2 | 0.9381324887223157 | 0.4890232744592855 | 0.601934664715668 | 0.9999971192244244 | 0.7277868481355678 | 3 |
| Midea Group Co., Ltd. | 14 | 51.3333333333333 | 1 | 1 | 0.444773489237885 | 0.4219990143068912 | 0.9773673422809854 | 3 |
| Mingshuo Computer (Suzhou) Co., Ltd. | 0.5 | 0.5 | 0.18241484628562907 | 0.4278695327024636 | 0.4198177004592919 | 0.4219990143068912 | 0.36184685290540625 | 1 |

| | | | | | | | | |
|---|---|---|---|---|---|---|---|---|
| Csg Electric Power Research Institute Co., Ltd. | 12.0666666666667 | 20.3333333333333 | 0.9998710574239954 | 1 | 0.8934528144007778 | 0.49454880229046416 | 0.993800419769849 | 3 |
| NARI Group Co., Ltd. | 0.75 | 0.45 | 0.5001370302919633 | 1 | 0.9730682357849149 | 1 | 0.9823107065787713 | 2 |
| Nr Electric Co., Ltd. | 3.33333333333333 | 7.7 | 0.8929264803996686 | 1 | 0.9693355304492586 | 0.6929262012459878 | 0.7664101480622485 | 2 |
| Nr Engineering Co., Ltd. | 2.83333333333333 | 5.5 | 0.663368062786774 | 0.4278695327024636 | 0.9657442113252727 | 0.5153934704296111 | 0.49520282558735257 | 1 |
| Nanjing Nanrui Information And Communication Technology Co., Ltd. | 9.53333333333333 | 3.2 | 0.9927619881530132 | 1 | 0.9701622249457315 | 0.9935937117287273 | 0.9290318803906827 | 2 |
| Nanjing Nuoyuan Medical Devices Co., Ltd. | 4 | 6 | 0.18241484628562907 | 0.4278695327024636 | 0.41833149742681786 | 0.4219990143068912 | 0.36184685290540625 | 1 |
| Nanjing Shengmingyuan Medicine Industry Co., Ltd. | 1 | 0 | 0.18241484628562907 | 0.4278695327024636 | 0.41833149742681786 | 0.4219990143068912 | 0.36184685290540625 | 1 |
| Nanjing Panda Electronics Company Limited | 0.5 | 0.333333333333333 | 0.9839146227189915 | 0.9995212583925209 | 0.4299410297126085 | 0.5045904592909234 | 0.625348266688536 | 3 |
| Nanjing Panda Mechatronics Instrument Technology Co., Ltd. | 0 | 0.333333333333333 | 0.663368062786774 | 0.4278695327024636 | 0.42566386361481257 | 0.5153934704296111 | 0.16947738966844986 | 3 |
| Nanjing Panda Information Industry Co., Ltd. | 0.5 | 0.333333333333333 | 0.8295080017104791 | 0.4278695327024636 | 0.42566386361481257 | 0.5001142013848573 | 0.361054784107187 | 3 |
| Nanjing Yuyue Software Technology Co., Ltd. | 2.2 | 4.11666666666667 | 0.9999999999979519 | 0.4278695327024636 | 0.4271000486954222 | 0.5000068383457262 | 0.5393853417673535 | 3 |
| Nari Group Corporation | 13.4277777777778 | 5.35 | 0.9381324887223157 | 1 | 0.9685637502805945 | 0.999999726345777 | 0.9449340680830806 | 2 |
| Nubia Technology Co., Ltd. | 27 | 132.5 | 0.06800820290420181 | 0.4278695327024636 | 0.41833149742681786 | 0.4219990143068912 | 0.36184685290540625 | 1 |
| Qi An Xin Technology Group Inc. | 12.5 | 3.5 | 0.26364124754974233 | 0.5617606422815072 | 0.4227607901018912 | 0.4219990143068912 | 0.6173125877472777 | 3 |
| Qizhi Software (Beijing) Co., Ltd. | 5 | 5 | 0.03664507312993 | 0.4278695327024636 | 0.4208031079855378 | 0.4219990143068912 | 0.36184685290540625 | 1 |

| | | | | | | | | |
|---|---|---|---|---|---|---|---|---|
| Qingdao Haier Multimedia Co., Ltd. | 0.5 | 0.5 | 0.036645073 12993 | 0.42786953 27024636 | 0.41833149742 681786 | 0.42199901 43068912 | 0.361846852 90540625 | 1 |
| Qingdao Haier Intelligent Home Appliance Technology Co., Ltd. | 1 | 13.5 | 0.482711703 02490723 | 0.42786953 27024636 | 0.42276079010 18912 | 0.80702935 51164697 | 0.477799173 4158749 | 1 |
| Samsung Electronics Co., Ltd. | 8.5 | 35 | 1 | 0.99999504 21389212 | 0.42994102971 26085 | 0.42199901 43068912 | 0.982010745 3255179 | 3 |
| Shandong Luneng Intelligence Technology Co., Ltd. | 2.5 | 8 | 0.263641247 54974233 | 0.42786953 27024636 | 0.83279461160 39252 | 0.99999711 92244244 | 0.730628574 1268843 | 2 |
| Shandong New Beiyang Information Technology Co., Ltd. | 4 | 3.5 | 0.350565379 52743944 | 0.42786953 27024636 | 0.41833149742 681786 | 0.42199901 43068912 | 0.361846852 90540625 | 1 |
| Shandong Zhengxin Medical Technology Co., Ltd. | 1 | 3.5 | 0.523770173 4645818 | 0.42786953 27024636 | 0.41833149742 681786 | 0.42199901 43068912 | 0.361846852 90540625 | 1 |
| Shanghai Datang Mobile Communications Equipment Co., Ltd. | 2 | 0 | 0.068008202 90420181 | 0.49987519 22541408 | 0.42129417787 23078 | 0.42199901 43068912 | 0.859868636 7957954 | 3 |
| Shanghai Hengguang Police Used Equipment Co., Ltd. | 0 | 0.5 | 0.068008202 90420181 | 0.42786953 27024636 | 0.42129417787 23078 | 0.42199901 43068912 | 0.361846852 90540625 | 1 |
| Shanghai Ic R&D Center Co., Ltd. | 8 | 2 | 0.182414846 28562907 | 0.42786953 27024636 | 0.41981770045 92919 | 0.42199901 43068912 | 0.361846852 90540625 | 1 |
| Shanghai KOSTAL - Huayang Automotive Electric Co., Ltd. | 2.5 | 2 | 0.116120659 89230397 | 0.42786953 27024636 | 0.41833149742 681786 | 0.42199901 43068912 | 0.361846852 90540625 | 1 |
| Shanghai Tianma Micro-Electronics Co., Ltd. | 6 | 43 | 0.116120659 89230397 | 0.42786953 27024636 | 0.42421739815 171033 | 0.42199901 43068912 | 0.361846852 90540625 | 1 |
| Nextvpu (Shanghai) Co., Ltd. | 5.3333333333 3333 | 3 | 0.428871494 58057516 | 0.42786953 27024636 | 0.42129417787 23078 | 0.93681907 5702284 | 0.396165852 7234138 | 1 |
| Saic General Motors Corp. Ltd. | 2 | 1.5 | 0.116120659 89230397 | 0.56176064 22815072 | 0.42276079010 18912 | 0.42199901 43068912 | 0.793097574 254146 | 3 |
| Eyecool Shenzheng Technology Co., Ltd. | 3.8333333333 3333 | 7.166666666 66667 | 0.428871494 58057516 | 0.42786953 27024636 | 0.42757646880 03926 | 0.93681907 5702284 | 0.000127742 00977858346 | 1 |
| Shenzhen Skyworth Digital Technology Co., Ltd. | 11 | 6 | 0.036645073 12993 | 0.42786953 27024636 | 0.41833149742 681786 | 0.42199901 43068912 | 0.361846852 90540625 | 1 |

| | | | | | | | | |
|---|---|---|---|---|---|---|---|---|
| Shenzhen Futaihong Precision Industrial Co., Ltd. | 1 | 1 | 0.26364124754974233 | 0.5617606422815072 | 0.42421739815171033 | 0.9999971192244244 | 0.836137888892724 | 3 |
| Shenzhen Power Supply Co., Ltd. | 32.8333333333333 | 16.3333333333333 | 0.5237701734645818 | 1 | 0.8725979037132321 | 0.9999999999999986 | 0.9567931517987001 | 2 |
| Shenzhen Mindray Scientific Co., Ltd. | 3.5 | 14 | 0.9999998713458631 | 0.4278695327024636 | 0.4212941778723078 | 0.4219990143068912 | 0.36184685290540625 | 3 |
| Shenzhen Mindray Bio-Medical Electronics Co., Ltd. | 13.5 | 72.5 | 0.9999999954746681 | 0.7116893099893431 | 0.42421739815171033 | 0.4219990143068912 | 0.4904475054254476 | 3 |
| First Dome Corp Telecom Co., Ltd. | 1.5 | 1 | 0.35056537952743944 | 0.4278695327024636 | 0.41833149742681786 | 0.4219990143068912 | 0.36184685290540625 | 1 |
| Shenzhen Guohua Optoelectronic Tech. Co., Ltd. | 0.5 | 0 | 0.18241484628562907 | 0.4278695327024636 | 0.4227607901018912 | 1 | 0.2862570260876587 | 1 |
| Shenzhen Huazhenglian Industrial Co., Ltd. | 3.5 | -4.44E-16 | 0.35056537952743944 | 0.4278695327024636 | 0.4212941778723078 | 0.9956524920387434 | 0.18659807662198596 | 1 |
| Shenzhen Comtop Information Technology Co., Ltd. | 0.5 | 0 | 0.5001370302919633 | 1 | 0.7668083410318259 | 1 | 0.9035337847404951 | 2 |
| Shenzhen Sayesmed Co., Ltd. | 0 | 3.5 | 0.06800820290420181 | 0.4278695327024636 | 0.6458966221932664 | 0.4219990143068912 | 0.36184685290540625 | 1 |
| Shenzhen Tencent Computer Systems Company Limited | 8.5 | 24.5 | 0.26364124754974233 | 1 | 0.8782458178400577 | 0.9999971192244244 | 0.8883396138702683 | 2 |
| Shenzhen yASUN Technology Co., Ltd. | 2.5 | 1.5 | 0.48271170302490723 | 0.4278695327024636 | 0.4198177004592919 | 0.4219990143068912 | 0.36184685290540625 | 1 |
| Shenzhen Yihua Times Technology Co., Ltd. | 1 | 4.3333333333333 | 0.9999998713458631 | 0.4278695327024636 | 0.4212941778723078 | 0.4544542497967531 | 0.18659807662195577 | 3 |
| Shenzhen Rc Intelligence Technology Co., Ltd. | 1 | 0 | 0.42887149458057516 | 0.9762061992732984 | 0.7636333485347768 | 0.9999899351919338 | 0.790914591478932 | 2 |
| Shenzhen Nubia Mobile Software Co., Ltd. | 0 | 1 | 0.06800820290420181 | 0.4278695327024636 | 0.41833149742681786 | 0.4219990143068912 | 0.36184685290540625 | 1 |
| Shenzhen Yihua Computer Co., Ltd. | 1.33333333333333 | 4.3333333333333 | 0.9999998713458631 | 0.4278695327024636 | 0.4212941778723078 | 0.4544542497967531 | 0.18659807662195577 | 3 |

| | | | | | | | | |
|---|---|---|---|---|---|---|---|---|
| China Energy Guohua (Beijing) Electric Power Research Institute Co., Ltd. | 1.33333333333333 | 0 | 0.48271170302490723 | 0.9999999999999996 | 0.438203118958126 | 0.9981235905421787 | 0.6423625215072293 | 1 |
| Getac Technology Corporation | 2.5 | 4.5 | 0.5237701734645818 | 0.4278695327024636 | 0.41833149742681786 | 0.4219990143068912 | 0.36184685290540625 | 1 |
| Shenxun Computer (Kunshan) Co., Ltd. | 2.5 | 4.5 | 0.5237701734645818 | 0.4278695327024636 | 0.41833149742681786 | 0.4219990143068912 | 0.36184685290540625 | 1 |
| Shijue Co., Ltd. | 0.5 | 0.75 | 0.5237701734645818 | 0.4278695327024636 | 0.4271000486954222 | 0.9999999999332949 | 0.49638658378036066 | 1 |
| Dawning Information Industry (Beijing) Corp., Ltd. | 0.333333333333333 | 0.833333333333333 | 0.5001370302919633 | 0.5617606422815072 | 0.4227607901018912 | 0.4219990143068912 | 0.6529329453172306 | 3 |
| Dawning Information Industry Co., Ltd. | 0.333333333333333 | 1.83333333333333 | 0.48271170302490723 | 0.5617606422815072 | 0.4227607901018912 | 0.4219990143068912 | 0.5148358226821336 | 3 |
| Sifang Jibao (Wuhan) Software Co., Ltd. | 1 | 0.5 | 0.35056537952743944 | 0.47937350499153036 | 0.42566386361481257 | 0.9999999999332949 | 0.4989447540709798 | 1 |
| Suzhou Jiashi Dadian Communication Information Technology Co., Ltd. | 1.5 | 5.5 | 0.8295080017104791 | 0.4998751922541408 | 0.4212941778723078 | 0.4219990143068912 | 0.49301906986136446 | 3 |
| Suzhou Tsingtech Microvision Electronic Technology Co., Ltd. | 3 | 0 | 0.26364124754974233 | 0.4278695327024636 | 0.41833149742681786 | 0.4219990143068912 | 0.36184685290540625 | 1 |
| Samsung Electronics Suzhou Computer Co., Ltd. | 4 | 9 | 0.9999999992510294 | 0.4278695327024636 | 0.42421739815171033 | 0.4219990143068912 | 0.36184685290540625 | 3 |
| Suzhou Medical Appliance Factory Ltd. | 2.2 | 3 | 0.9988586920675357 | 0.4278695327024636 | 0.4271000486954222 | 0.6929262012459878 | 0.4976257575173691 | 3 |
| Suzhou Yuyue Medical Technology Co., Ltd. | 2.2 | 3.45 | 0.999989562420184 | 0.4278695327024636 | 0.4271000486954222 | 0.5624327155406912 | 0.5000000806639495 | 3 |
| Tencent Technology (Shenzhen) co., Ltd. | 268.5 | 1844.5 | 1 | 1 | 0.9055382116411006 | 0.4312243556496435 | 0.8951813696423605 | 3 |
| Tencent Cloud Computing (Beijing) Co., Ltd. | 7 | 12 | 0.9999999999999927 | 0.4278695327024636 | 0.7530254597044085 | 0.4219990143068912 | 0.36184685290540625 | 3 |
| TianJin ZhongWei Aerospace Data System Technology Co., Ltd. | 2 | 1.5 | 0.35056537952743944 | 0.4278695327024636 | 0.7422326890149382 | 0.4219990143068912 | 0.36184685290540625 | 1 |

| | | | | | | | | |
|---|---|---|---|---|---|---|---|---|
| Tianma Microelectronics Co., Ltd. | 0 | 3 | 0.48271170302490723 | 0.999943759654791 | 0.4299410297126085 | 0.8070293551164697 | 0.983291802624848 | 3 |
| Nuctech Company Limited | 14.5 | 13.5 | 0.5001370302919633 | 1 | 0.8711558229788038 | 0.6797196065514214 | 0.565195126185651 | 1 |
| Weihai New Beiyang Digital Technology Co., Ltd. | 0.5 | 2.5 | 0.35056537952743944 | 0.4278695327024636 | 0.41833149742681786 | 0.4219990143068912 | 0.36184685290540625 | 1 |
| Wistron Infocomm (Zhongshan) Corporation | 0 | 0.5 | 0.5001370302919633 | 0.4278695327024636 | 0.4227607901018912 | 0.4219990143068912 | 0.36184685290540625 | 1 |
| Wistron Corporation | 10 | 44.5 | 0.999600056775284 | 0.9853409345508982 | 0.4271000486954222 | 0.4219990143068912 | 0.9635900490272601 | 3 |
| Wuxi Xinje Electric Co., Ltd. | 1 | 0 | 0.26364124754974233 | 0.4278695327024636 | 0.4212941778723078 | 0.4219990143068912 | 0.36184685290540625 | 1 |
| Wuhu Midea Kitchen and Bath Appliances Mfg. Co., Ltd. | 2.5 | 1.5 | 0.18241484628562907 | 0.4278695327024636 | 0.43227589678616174 | 0.4219990143068912 | 0.36184685290540625 | 1 |
| Xi'an ASN Technology Group Company | 3.5 | 5 | 0.18241484628562907 | 0.4278695327024636 | 0.8239584206399964 | 0.4219990143068912 | 0.36184685290540625 | 1 |
| Xi'an Cetc-Cetc Radar Technology Collaborative Innovation Research Institute Co., Ltd. | 4.5 | 3 | 0.35056537952743944 | 0.4278695327024636 | 0.4198177004592919 | 0.4219990143068912 | 0.36184685290540625 | 1 |
| Xilinmen Furniture Co., Ltd. | 5.5 | 8 | 0.18241484628562907 | 0.4278695327024636 | 0.41833149742681786 | 0.4219990143068912 | 0.36184685290540625 | 1 |
| Xiaomi Inc. | 0.5 | 26.5 | 0.5237701734645818 | 1 | 0.4341219910300546 | 0.4219990143068912 | 0.9693301085718816 | 3 |
| New Founder Holding Development Co., Ltd. | 0 | 6.66666666666667 | 1 | 1 | 0.4395406913639146 | 0.45184139386377264 | 0.6966422119859421 | 3 |
| Xingyun (Xiamen) Medical Technology Co., Ltd. | 0 | 1 | 0.18241484628562907 | 0.4278695327024636 | 0.41833149742681786 | 0.4219990143068912 | 0.36184685290540625 | 1 |
| Xuchang Xj Software Technelogy Co., Ltd. | 0.666666666666667 | 1 | 0.9998710574239954 | 0.48096015718652985 | 0.9670642400094723 | 0.9354794384506699 | 0.7799660947937053 | 2 |
| Xj Electric Co., Ltd. | 1.16666666666667 | 0.75 | 0.9839146227189915 | 0.4278695327024636 | 0.9666031687547725 | 0.9945898205095249 | 0.6985398493341928 | 2 |

| | | | | | | | | |
|---|---|---|---|---|---|---|---|---|
| Xj Group Corporation | 0.916666666666667 | 1.95 | 0.9999973793726163 | 1 | 0.9745414123437987 | 0.9258187601539084 | 0.849063720348447 | 2 |
| Interface Optoelectronics (Sz) Co., Ltd. | 3.33333333333333 | 3.16666666666667 | 0.8929264803996686 | 0.4278695327024636 | 0.4212941778723078 | 0.49912663467057705 | 0.6928530807646311 | 3 |
| Interface Technology (Chengdu) Co., Ltd. | 3.66666666666667 | 2.83333333333333 | 0.5001370302919633 | 0.4278695327024636 | 0.4212941778723078 | 0.6797196065514214 | 0.06778902943310201 | 1 |
| Yi Bao Internet Medical Information Technology (Beijing) Co., Ltd. | 2.5 | 6 | 0.18241484628562907 | 0.4278695327024636 | 0.5804023915385799 | 0.4219990143068912 | 0.36184685290540625 | 1 |
| Inventec Appliances (Jiangning) corporation | 0.5 | 3.5 | 0.11612065989230397 | 0.4278695327024636 | 0.4212941778723078 | 0.4219990143068912 | 0.36184685290540625 | 1 |
| Inventec Appliances (Shanghai) Co., Ltd. | 3.5 | 2.25 | 0.5237701734645818 | 0.4278695327024636 | 0.4227607901018912 | 0.5922258094371584 | 0.3018247709479677 | 1 |
| Inventec Appliances (Shanghai) corporation | 3.5 | 2.25 | 0.5237701734645818 | 0.4278695327024636 | 0.4227607901018912 | 0.5922258094371584 | 0.3018247709479677 | 1 |
| Inventec Appliances Corporation | 2.33333333333333 | 5.91666666666667 | 0.7502368209118209 | 0.5617606422815072 | 0.42421739815171033 | 0.5037075637825447 | 0.5688781637078492 | 3 |
| Dongguan Solution 33 Electronic Technology Co., Ltd. | 0.5 | 0.75 | 0.663368062786774 | 0.4278695327024636 | 0.4271000486954222 | 0.9999704258833632 | 0.5052687360991258 | 1 |
| Solution 33 Electronic Technology Co., Ltd. Taiwan Branch | 0.5 | 0.75 | 0.663368062786774 | 0.4278695327024636 | 0.4271000486954222 | 0.9999704258833632 | 0.5052687360991258 | 1 |
| Yingtesheng Technology Co., Ltd. | 2.33333333333333 | 2.66666666666667 | 0.48271170302490723 | 0.4278695327024636 | 0.4212941778723078 | 0.8070293551164697 | 0.03148374084712985 | 1 |
| Inventec (Chongqing) Corporation | 3 | 0 | 0.663368062786774 | 0.4278695327024636 | 0.4227607901018912 | 0.5153934704296111 | 0.0008913889384944899 | 3 |
| Inventec Corporation | 5.5 | 4 | 0.9999999992510294 | 0.47937350499153036 | 0.42421739815171033 | 0.47093369308169414 | 0.46804993788147203 | 3 |
| Inventec (Pudong) Technology Corporation | 5.5 | 4 | 0.9999999992510294 | 0.47937350499153036 | 0.42421739815171033 | 0.47093369308169414 | 0.46804993788147203 | 3 |
| Yulong Computer Telecommunication Scientific (Shenzhen) Co., Ltd. | 3.33333333333333 | 43 | 0.06800820290420181 | 0.4278695327024636 | 0.41833149742681786 | 0.4219990143068912 | 0.36184685290540625 | 1 |

| | | | | | | | | |
|---|---|---|---|---|---|---|---|---|
| Changsha Xiangji Haidun Technology Co., Ltd. | 0.5 | 2 | 0.036645073 12993 | 0.42786953 27024636 | 0.41833149742 681786 | 0.42199901 43068912 | 0.361846852 90540625 | 1 |
| Zhejiang Geely Holding (Group) Co., Ltd. | 25.333333333 3333 | 12.33333333 33333 | 0.938132488 7223157 | 0.94726994 81686714 | 0.42710004869 54222 | 0.49613731 245029824 | 0.615115015 9696382 | 3 |
| Zhejiang Geely Automobile Research Institute Co., Ltd. | 9.5 | 4 | 0.750236820 9118209 | 0.42786953 27024636 | 0.42276079010 18912 | 0.42199901 43068912 | 0.361846852 90540625 | 3 |
| Zhejiang Somnic Technology Co., Ltd. | 0.5 | 0 | 0.182414846 28562907 | 0.42786953 27024636 | 0.41833149742 681786 | 0.42199901 43068912 | 0.361846852 90540625 | 1 |
| Cheng Uei Precision Industry Co., Ltd. | 0.5 | 0 | 0.350565379 52743944 | 0.71168930 99893431 | 0.42421739815 171033 | 0.42199901 43068912 | 0.948062067 9899794 | 3 |
| China Nuclear Power Technology Research Institute Co., Ltd. | 0.5833333333 33333 | 0.2 | 0.992761988 1530132 | 1 | 0.67159241085 0622 | 0.98429149 39856674 | 0.766482093 5855228 | 3 |
| China Electric Power Research Institute | 26.866666666 6667 | 45.26666666 66667 | 1 | 1 | 0.98348279771 81487 | 0.56937933 80055954 | 0.980337758 3141337 | 2 |
| China Guangdong Nuclear Power Group Co., Ltd. | 4.4357142857 1429 | 0.949999999 999999 | 0.999999995 4746681 | 0.99997069 27241899 | 0.60678133290 95408 | 0.82233582 50427348 | 0.856729594 6530663 | 3 |
| Cgn Power Co., Ltd. | 4.4357142857 1429 | 2.783333333 33333 | 1 | 1 | 0.68420972971 84894 | 0.60270551 18846739 | 0.967343806 9711436 | 3 |
| China Railway Corporation | 7.5833333333 3333 | 4.166666666 66667 | 0.750236820 9118209 | 1 | 0.45965143088 575416 | 0.99996084 90508354 | 0.995283207 633155 | 3 |
| China National Offshore Oil Corp. | 5.8333333333 3333 | 0.5 | 0.068008202 90420181 | 0.49987519 22541408 | 0.42129417787 23078 | 0.42199901 43068912 | 0.859868636 7957954 | 3 |
| China United Network Communication Group Co., Ltd. | 54.5 | 83.5 | 0.116120659 89230397 | 0.42786953 27024636 | 0.41833149742 681786 | 0.42199901 43068912 | 0.361846852 90540625 | 1 |
| China Southern Power Grid Company Limited | 21 | 59.36666666 66667 | 0.750236820 9118209 | 1 | 0.88910826269 34433 | 0.95888490 819231 | 0.987612135 4422975 | 2 |
| Commercial Aircraft Corporation Of China, Ltd. | 3.3333333333 3333 | 3.5 | 0.263641247 54974233 | 0.49987519 22541408 | 0.42129417787 23078 | 0.42199901 43068912 | 0.837439997 9071557 | 3 |
| China Shenhua Energy Company Limited | 4.5 | 0 | 0.582568989 5709085 | 1 | 0.44477348923 7885 | 0.97290100 24545853 | 0.990204862 7282038 | 3 |

| | | | | | | | | |
|---|---|---|---|---|---|---|---|---|
| China Petroleum and Chemical Corporation | 9 | 13.6666666666667 | 0.9381324887223157 | 1 | 0.44981217355729164 | 0.7708212268502525 | 0.9972767283986467 | 3 |
| China Petroleum And Chemical Corporation Qingdao Security Engineering Research Institute | 1 | 0 | 0.18241484628562907 | 0.4278695327024636 | 0.43549363706655925 | 0.4219990143068912 | 0.36184685290540625 | 1 |
| Petroleum Exploration and Development Research Institute of Sinopec Corporation | 0.5 | 1.5 | 0.11612065989230397 | 0.4278695327024636 | 0.43549363706655925 | 0.4219990143068912 | 0.36184685290540625 | 1 |
| Bgp Inc., China National Petroleum Corporation | 0 | 1 | 0.26364124754974233 | 0.4278695327024636 | 0.4327392518864463 | 0.4219990143068912 | 0.36184685290540625 | 1 |
| Petrochina Company Limited | 8.5 | 27.5 | 0.03664507312993 | 0.4278695327024636 | 0.41833149742681786 | 0.4219990143068912 | 0.36184685290540625 | 1 |
| China National Petroleum Corporation | 2.5 | 5.33333333333333 | 0.9927619881530132 | 1 | 0.4460516741361321 | 0.5009056295357938 | 0.9977149044698586 | 3 |
| China Academy Of Railway Sciences | 15.75 | 2.33333333333333 | 0.5825689895709085 | 1 | 0.45385703634421676 | 0.9999704258833632 | 0.9273733184515753 | 3 |
| China Mobile Communications Corporation | 25.6666666666667 | 143.166666666667 | 0.5825689895709085 | 1 | 0.8520030400022471 | 0.4219990143068912 | 0.9956732192600783 | 3 |
| Zhongchao Hismart Information Technology Co., Ltd. | 1 | 1 | 0.35056537952743944 | 0.9853409345508982 | 0.4271000486954222 | 0.4219990143068912 | 0.9855129376079726 | 3 |
| Gree Electric Appliances, Inc. Of Zhuhai | 101 | 280.5 | 0.35056537952743944 | 0.9999950421389212 | 0.4299410297126085 | 0.4219990143068912 | 0.9931224610618246 | 3 |
| Zhuhai Kingsoft Office Software Co., Ltd. | 1 | 8.16666666666667 | 0.999600056775284 | 0.4278695327024636 | 0.4212941778723078 | 0.4711833197143965 | 0.232103636356506 | 3 |
| Zhuhai Kingsoft Digital Network Technology Co., Ltd. | 4.5 | 2.5 | 0.48271170302490723 | 0.7116893099893431 | 0.42421739815171033 | 0.4219990143068912 | 0.9680032067429901 | 3 |
| Zhuhai Unitech Power Technology Co., Ltd. | 13.3333333333333 | 42 | 0.35056537952743944 | 1 | 0.967368724715669 | 1 | 0.9201764156435972 | 2 |
